# Pore Engineering for One-Step Ethylene Purification from a Three-Component Hydrocarbon Mixture

**DOI:** 10.1021/jacs.0c11247

**Published:** 2021-01-13

**Authors:** Baoyong Zhu, Jian-Wei Cao, Soumya Mukherjee, Tony Pham, Tao Zhang, Teng Wang, Xue Jiang, Katherine A. Forrest, Michael J. Zaworotko, Kai-Jie Chen

**Affiliations:** ‡ Key Laboratory of Special Functional and Smart Polymer Materials of Ministry of Industry and Information Technology, Xi’an Key Laboratory of Functional Organic Porous Materials, School of Chemistry and Chemical Engineering, Northwestern Polytechnical University, Xi’an, Shaanxi 710072, P.R. China; § Bernal Institute, Department of Chemical Sciences, University of Limerick, Limerick V94 T9PX, Republic of Ireland; ∥ School of Chemistry and Chemical Engineering, Dezhou University, Dezhou 253023, P.R. China; ⊥ Department of Chemistry, University of South Florida, 4202 East Fowler Avenue, CHE205, Tampa, Florida 33620-5250, United States

## Abstract

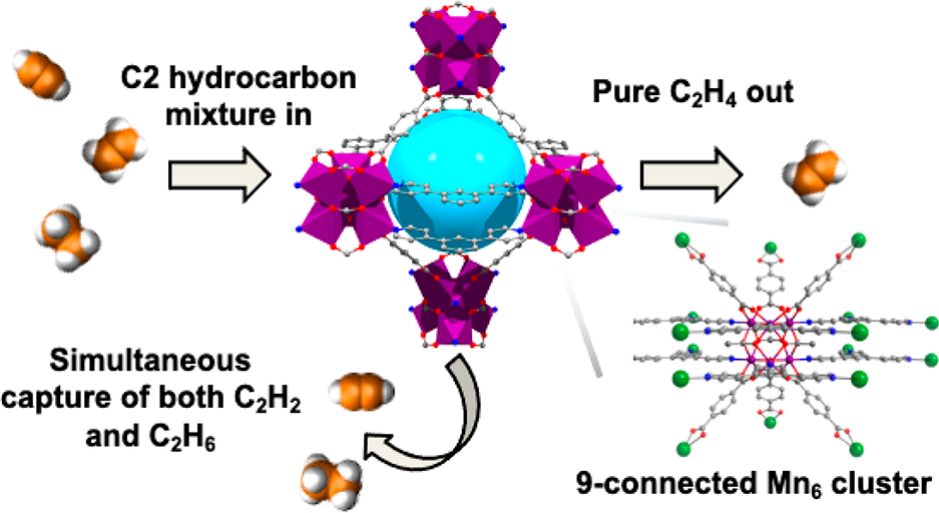

Ethylene production from C2 hydrocarbon mixtures through one separation step is desirable but challenging because of the similar size and physical properties of acetylene, ethylene, and ethane. Herein, we report three new isostructural porous coordination networks (**NPU-1**, **NPU-2**, **NPU-3**; NPU represents Northwestern Polytechnical University) that are sustained by 9-connected nodes based upon a hexanuclear metal cluster of composition [Mn_6_(μ_3_-O)_2_(CH_3_COO)_3_]^6+^. **NPU-1/2/3** exhibit a dual cage structure that was systematically fine-tuned in terms of cage size to realize selective adsorption of C_2_H_2_ and C_2_H_6_ over C_2_H_4_. Dynamic breakthrough experiments demonstrated that **NPU-1** produces ethylene in >99.9% purity from a three-component gas mixture (1:1:1 C_2_H_2_/C_2_H_4_/C_2_H_6_). Molecular modeling studies revealed that the dual adsorption preference for C_2_H_2_ and C_2_H_6_ over C_2_H_4_ originates from (a) strong hydrogen-bonding interactions between electronegative carboxylate O atoms and C_2_H_2_ molecules in one cage and (b) multiple non-covalent interactions between the organic linkers of the host network and C_2_H_6_ molecules in the second cage.

## Introduction

Ethylene (C_2_H_4_) is one of the most important industrial products, with production levels of >150 million tons in 2016.^1^ The energy footprint needed to purify ethylene and propylene (C_3_H_6_), another high-volume product, means that they collectively account for ca. 0.3% of global energy.^2^ Currently, C_2_H_4_ is mainly produced by steam-cracking reaction of carbon-included feedstocks, and high-purity ethylene is afforded by energy-intensive separation processes of downstream C2 hydrocarbon gas mixtures.^3^ Acetylene (C_2_H_2_) is first removed by catalytic hydrogenation using noble metal catalysts at high temperature and pressure, whereas ethane is later separated from C_2_H_4_ by cryogenic distillation. A transition to a more energy-efficient separation technology with a much lower energy footprint and simplified separation process (e.g., simultaneous removal of C_2_H_2_ and C_2_H_6_ in one step) for ethylene production would be of societal relevance.

Physisorption-based separation processes hold the promise for greatly reducing energy consumption of gas separation, thanks to the low regeneration temperature and fast sorption kinetics typical of physisorbents.^4^ Porous coordination networks based upon metal–organic materials (MOMs),^5^ also known as metal–organic frameworks (MOFs)^6,7^ and porous coordination polymers (PCPs),^8^ have emerged as promising C2 light hydrocarbon physisorbents due to their amenability to exquisite control of pore shape and chemistry through reticular chemistry and crystal engineering strategies.^9,10^ In this context, recent progress has been made with respect to binary separations such as C_2_H_2_/C_2_H_4_ and C_2_H_6_/C_2_H_4_.^11−32,32^ Simultaneous removal of both C_2_H_2_ and C_2_H_6_ in a single step would simplify the purification of C_2_H_4_ but remains a challenge for a single physisorbent. This is because the quadrupole moment and kinetic diameter for C_2_H_4_ (1.5 × 10^–26^ esu cm^2^ and 4.1 Å) sit between those of C_2_H_2_ (7.2 × 10^–26^ esu cm^2^ and 3.3 Å) and C_2_H_6_ (0.65 × 10^–26^ esu cm^2^ and 4.4 Å). These physicochemical properties exacerbate the challenge for one-step C_2_H_4_ production from C_2_H_2_ and C_2_H_6_ by either molecular sieving or thermodynamic selectivity. Thus far, we are aware of only three literature reports of C_2_H_4_ production from a C_2_H_2_–C_2_H_4_–C_2_H_6_ mixture in a one-step process. In 2018,^33^ simultaneous trapping of C_2_H_2_ and C_2_H_6_ was realized by the MOF **TJT-100** thanks to the hierarchy of weak sorbent–sorbate interactions. In 2019,^34^ we introduced the concept of synergistic sorbent separation technology (SSST) to enable high-purity ethylene production from the ternary mixture (C_2_H_2_–C_2_H_4_–C_2_H_6_) and quaternary mixture (C_2_H_2_–C_2_H_4_–C_2_H_6_–CO_2_) by using tandem packing of three MOMs in a fixed-bed sorbent. In SSST, three benchmark sorbents (**SIFSIX-3-Ni**, **TIFSIX-2-Cu-i**, and **Zn-atz-ipa**) were selected for CO_2_, C_2_H_2_, and C_2_H_6_ removal, respectively. Most recently,^35^ a Th-azole network (**Azole-Th-1**) enabled selective adsorption of ethane and acetylene over ethylene. The discovery of new physisorbents that enable selective adsorption of C_2_H_2_ and C_2_H_6_ over C_2_H_4_ is timely not just to address the practical utility of such sorbents but also to advance our understanding of C_2_H_2_, C_2_H_4_, and C_2_H_6_ binding sites in porous materials.

**Scheme 1:**
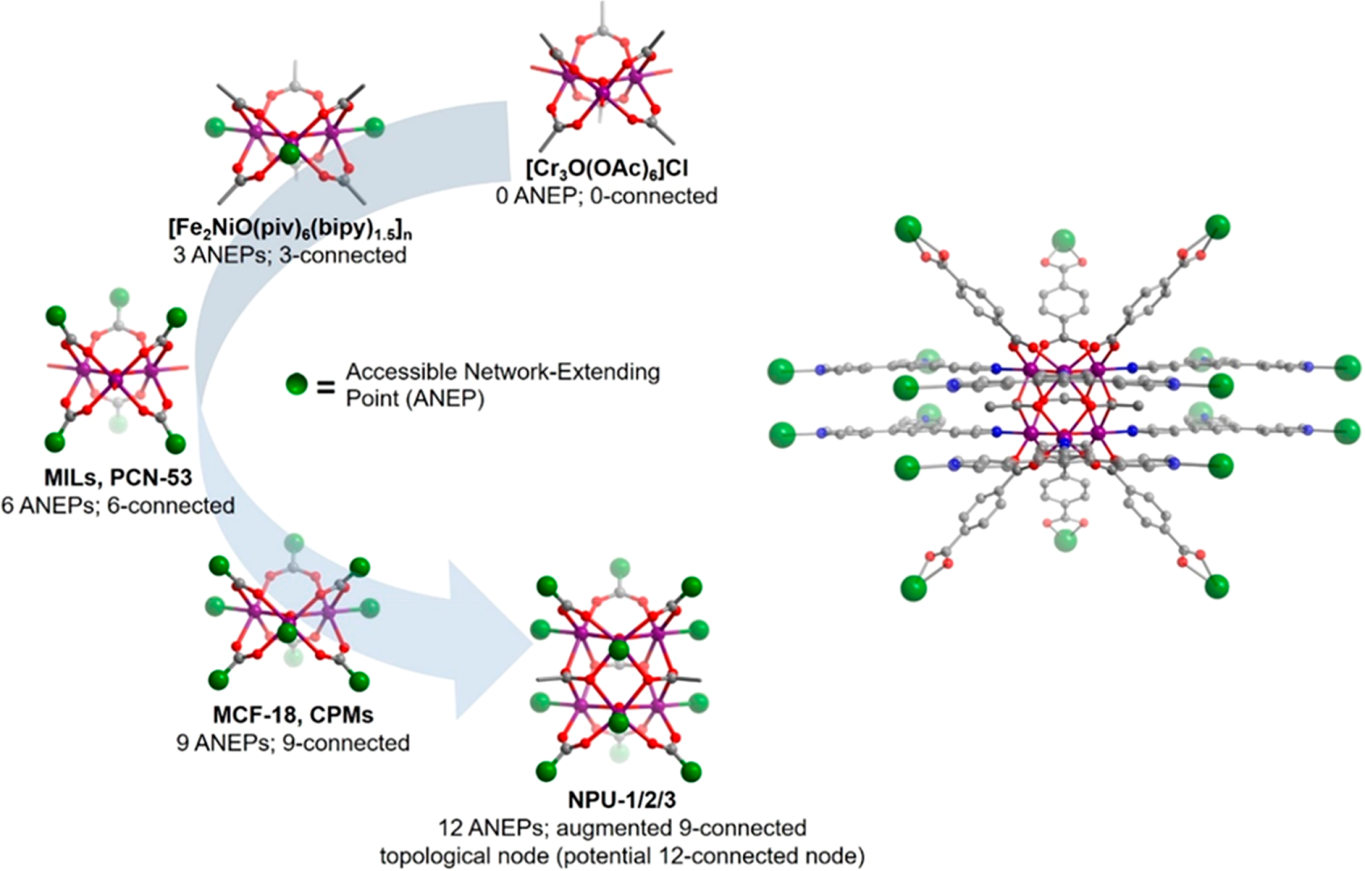
M_3_(μ_3_-O) Metal Clusters Are Well Studied and Have Previously Been Used to Construct 3-, 6-, or 9-Connected Porous Coordination Networks (Left); Augmented 9-Connected Networks Are Reported Herein from a New Mn_6_(μ_3_-O)_2_ Cluster (Right)

In this contribution, we introduce the new hexanuclear Mn cluster [Mn_6_(μ_3_-O)_2_(CH_3_COO)_3_]^6+^ and its use as a building block for three isostructural 9-connected (9-c) MOMs with varying pore size. The hexanuclear metal cluster can be viewed as a fusion of two well-known trinuclear metal clusters bridged by three acetate anions. The Mn_6_ cluster reported herein has the potential to sustain 12-c networks, whereas the trinuclear parent typically serves as a 3-c, 6-c, or 9-c node (Scheme 1).^36−41^ The linking of the Mn_6_ cluster by rigid dicarboxylate linker ligands (BDC = 1,4-benzenedicarboxylate; NPDC = naphthalene-2,6-dicarboxylate; BPDC = biphenyl-4,4′-dicarboxylate) and the 3-connected pyridyl-based tritopic ligand 2,4,6-tris(4-pyridyl)pyridine (Tripp) afforded three isostructural MOMs (**NPU-1**, **NPU-2**, and **NPU-3**; NPU = Northwestern Polytechnical University) with tunable pore size and pore chemistry. We report herein the C2 sorption and separation properties of these new sorbents.

## Results and Discussion

Single crystals of **NPU-1**, **NPU-2**, and **NPU-3** were harvested following solvothermal reaction of manganese acetate tetrahydrate, Tripp, and a dicarboxylic acid ligand (H_2_BDC, H_2_NPDC, or H_2_BPDC) in *N*,*N*-dimethylacetamide (DMA) at 373 K. Single-crystal structure analysis revealed that the three materials are isostructural 3D networks based on the aforementioned hexanuclear Mn_6_ cluster. The novel Mn_6_ cluster can be regarded as the result of fusion of two M_3_(μ_3_-O) clusters through bridging acetate anions. Each manganese cation adopts an octahedral geometry through coordination with two oxygen atoms from two dicarboxylate ligands, two oxygen atoms from acetate anions, one μ_3_-O atom, and one nitrogen atom from Tripp (Supplementary Figure 1). Overall charge neutrality of these networks requires mixed-valence Mn^III^ and Mn^II^ cations, which is supported by X-ray photoelectron spectroscopy (XPS) analysis (Supplementary Figure 2) and suggests electron delocalization. Unsuccessful ion-exchange experiments with charged dye molecules were conducted upon **NPU-1** and **NPU-3**, indicating an absence of extra-framework counterions (Supplementary Figure 3). Such dye exchange experiments have been utilized by others to verify the charge state of host coordination networks.^45^ The general formula for **NPU-1/2/3** is Mn_5_^II^Mn^III^(μ_3_-O)_2_(CH_3_COO)_3_(Tripp)_2_(L)_3_ (L = BDC^2–^/NPDC^2–^/BPDC^2–^ for **NPU-1/2/3**, respectively).

**Figure 1:**
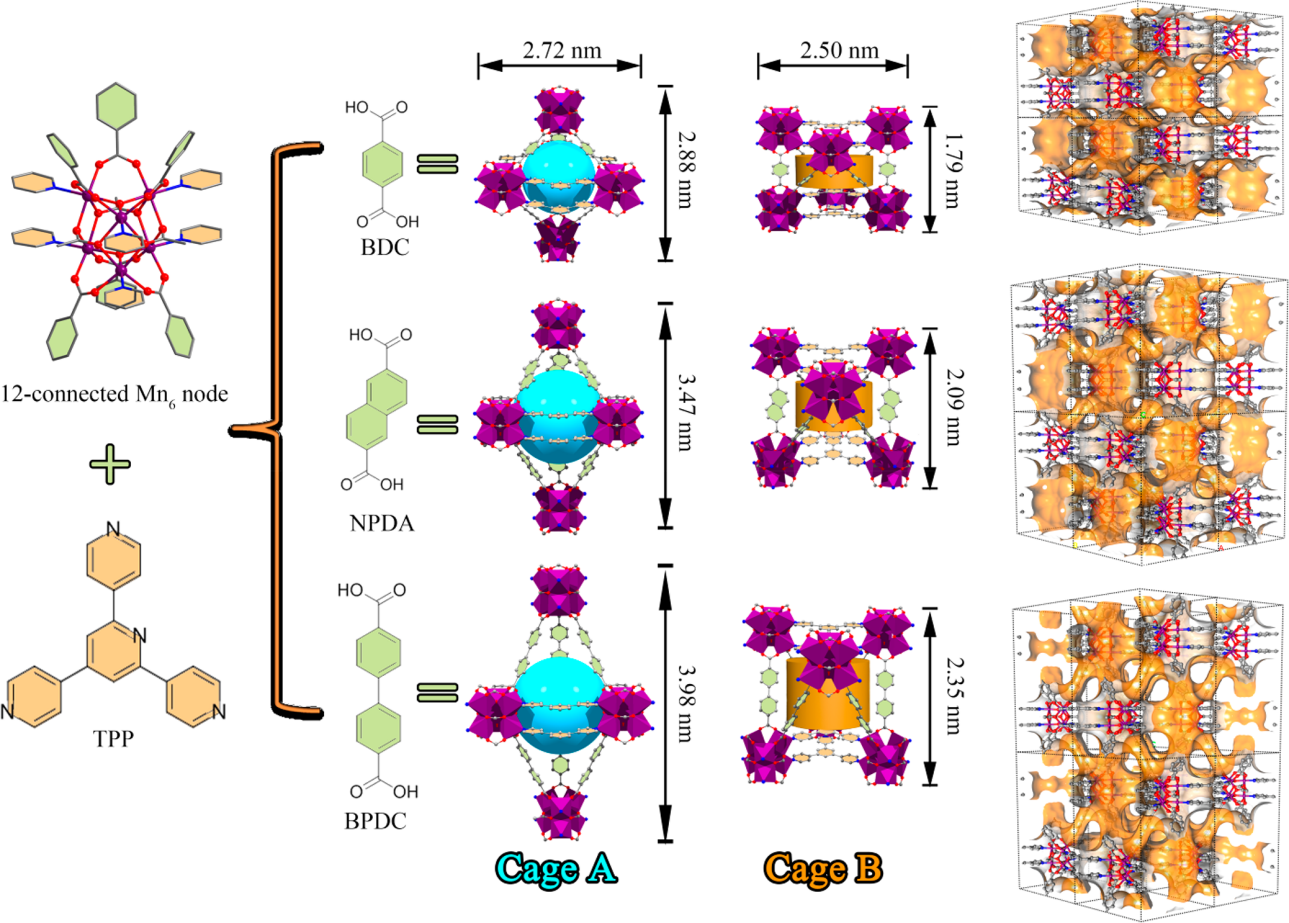
**NPU-1/2/3** are isostructural coordination networks that are based upon assembly of Mn_6_ clusters, Tripp ligands, and different dicarboxylate linker ligands. The three-dimensional pore structures comprise two distinct cages.

M_3_(μ_3_-O) clusters are normally connected by three, six, or nine organic linker ligands, but the Mn_6_ cluster in **NPU-1/2/3** is bonded to 12 linker ligands (six dicarboxylate ligands and six Tripp ligands). The potential for rare 12-c nodes exists,^5,42−44^ but pairs of Tripp ligands serve as parallel 3-c nodes that connect to the same adjacent Mn_6_ cluster. Therefore, the Mn_6_ cluster should be viewed as a 9-c node, and **NPU-1/2/3** are classified as 3,9-connected networks with **pacs** topology^46^ (Supplementary Figure 4). A similar “double cross-linking” situation occurred in the metal–organic polyhedron of formula [Cu_2_(bdc)_2_]_12_ (bdc = 1,3-benzenedicarboxylate). In this case, the 24 accessible connection points can serve as 6-c or 24-c nodes to build augmented **pcu** or **rht** networks.^47,48^ Notably, there are dual cages (A and B) in **NPU-1/2/3** (Figure 1). Cage A has a trigonal bipyramidal shape, as it is surrounded by five metal clusters, six dicarboxylate ligands, and six Tripp ligands. Whereas the equatorial dimension of Cage A is consistent in **NPU-1/2/3** (0.79 nm after subtracting the van der Waals radii of the nearest atoms), the axial dimension increases with the length of the dicarboxylate linker ligands (1.26, 1.77, and 2.24 nm for **NPU-1/2/3**, respectively). Cage B exhibits a distorted triangular prismatic shape, as it is formed from six adjacent Mn_6_ clusters, six dicarboxylate ligands, and four parallel Tripp ligands. The axial dimension in cage B varies from 0.38 to 0.66 to 0.91 nm for **NPU-1/2/3**, respectively. Each cage A is connected to six cage B’s in a face-shared manner, and vice versa. The triangular interconnecting pore windows between these cages, which are surrounded by one Tripp ligand and two carboxylate ligands, exhibit aperture sizes of 0.48 nm for **NPU-1**, 0.6 nm for **NPU-2**, and 0.78 nm for **NPU-3**. As revealed below, the dual cage nature of **NPU-1/2/3** is crucial to their selective adsorption of C_2_H_2_ and C_2_H_6_ over C_2_H_4_. The porosity of **NPU-1**, **NPU-2**, and **NPU-3** as calculated by PLATON^49^ was found to be 50.8%, 56.8%, and 62.1%, respectively.

**Figure 2:**
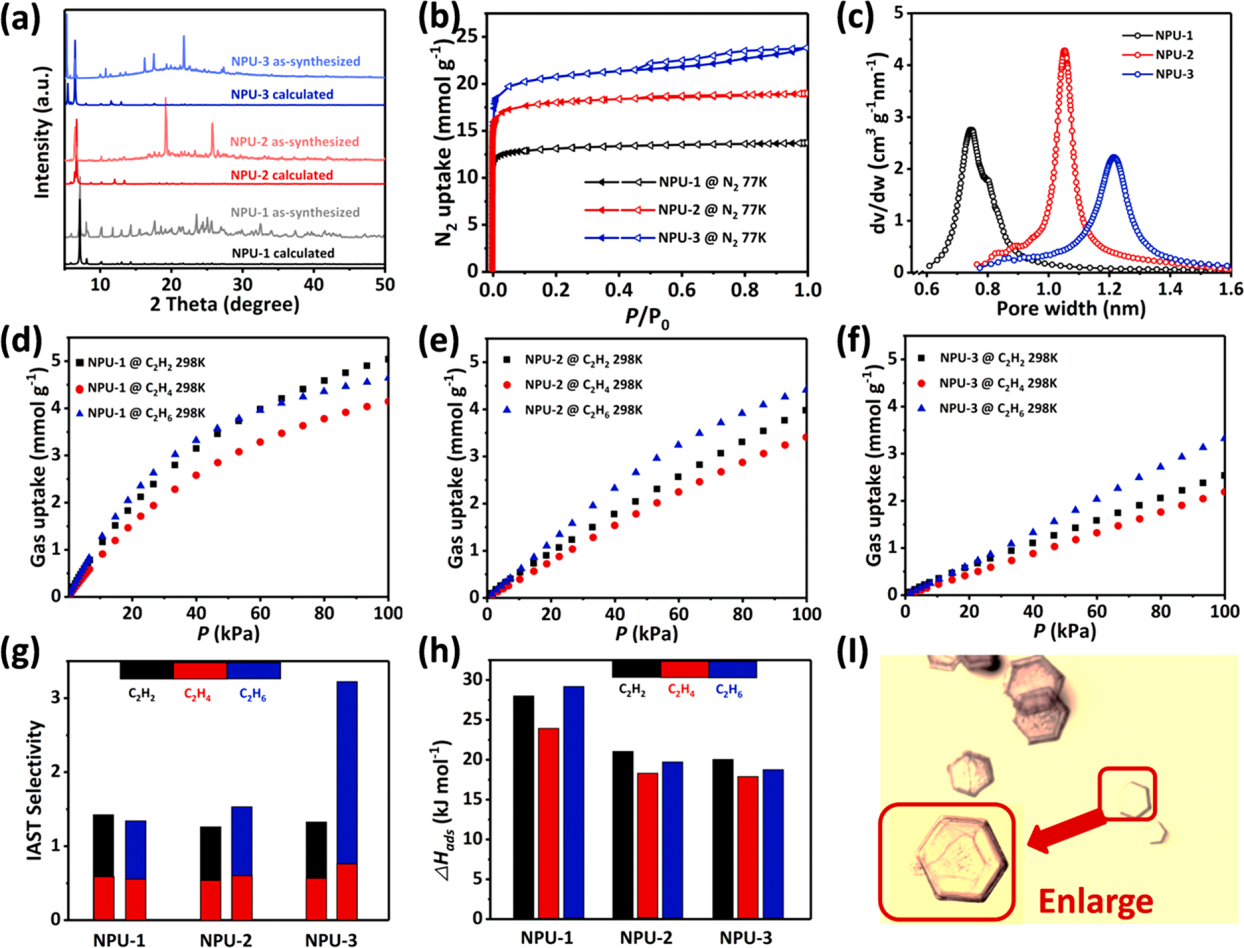
PXRD patterns and sorption data for **NPU-1**, **NPU-2**, and **NPU-3**. (a) Experimental and calculated PXRD patterns. (b) N_2_ sorption isotherms at 77 K. (c) Calculated pore-size distribution from 77 K N_2_ sorption data. (d) C2 gas sorption isotherms of **NPU-1**, (e) **NPU-2**, and (f) **NPU-3** at 298 K. (g) IAST selectivity of C_2_H_6_/C_2_H_4_ (1:1 ratio) and C_2_H_2_/C_2_H_4_ (1:1 ratio) at 298 K and 100 kPa. (h) C2 gas adsorption enthalpies at low loading. (i) Optical microscope image of **NPU-1** crystals.

Bulk purity of **NPU-1/2/3** was confirmed by powder X-ray diffraction (PXRD, Figure 2a and Supplementary Figures 6–8). Thermogravimetric analysis (TGA) of as-synthesized and CH_2_Cl_2_-exchanged samples revealed that the DMA solvent in the as-synthesized phase can be fully exchanged with CH_2_Cl_2_ and that **NPU-1/2/3** are thermally stable until 573 K (Supplementary Figures 9–11). N_2_ sorption experiments were conducted at 77 K to establish permanent microporosity, and **NPU-1/2/3** each exhibited reversible type-I adsorption isotherms. By assuming pore filling by liquid N_2_ at 77 K and 100 kPa, the pore volumes calculated from N_2_ uptake at 100 kPa are 0.47 for **NPU-1**, 0.66 for **NPU-2**, and 0.77 cm^3^ g^–1^ for **NPU-3**. These values match well with the values of 0.53 for **NPU-1**, 0.68 for **NPU-2**, and 0.84 cm^3^ g^–1^ for **NPU-3** calculated from the respective crystal structures. These findings are consistent with the absence of counterions in the pore channels of **NPU-1/2/3**. Langmuir and Brunauer–Emmett–Teller (BET) surface areas were calculated to be 1557 and 1396, 1844 and 1580, and 2133 and 1834 m^2^ g^–1^ for **NPU-1/2/3**, respectively. Horvath–Kawazoe (pore geometry: cylinder) and NLDFT model (pore geometry: slit)-based pore size distribution analyses were conducted upon the corresponding N_2_ sorption isotherms (Figure 2c and Supplementary Figure 12). A gradually increasing trend in pore size distribution was noticed upon increasing the length of the dicarboxylate ligands (peak position: 0.74 nm in **NPU-1**, 1.05 nm in **NPU-2**, and 1.22 nm in **NPU-3**), in agreement with the crystal structures.

The microporosity of **NPU-1/2/3** prompted us to collect pure gas sorption isotherms at 273 and 298 K for C_2_H_2_, C_2_H_4_, and C_2_H_6_ up to 100 kPa (Figure 2d–f, Supplementary Figures 13–15). **NPU-1** is the sorbent with the smallest pore size and exhibited the largest adsorption capacity for C_2_H_2_, C_2_H_4_, and C_2_H_6_, followed by **NPU-2** (intermediate pore size) and **NPU-3** (largest pore size). That the gas uptake at ambient temperatures was observed to increase with decreasing pore size is expected for microporous (<2 nm pores) materials.^50^ Notably, at 298 K, **NPU-1** was found to exhibit higher uptakes of C_2_H_2_ (5.1 mmol g^–1^) and C_2_H_6_ (4.5 mmol g^–1^) than C_2_H_4_ (4.2 mmol g^–1^) at 100 kPa. This trend is maintained at relatively low pressures (0–30 kPa), implying selective adsorption of C_2_H_2_ and C_2_H_6_ vs C_2_H_4_ across the range of loading in **NPU-1**. Despite lower C_2_H_2_ (4.03 and 2.58 mmol g^–1^, respectively), C_2_H_4_ (3.45 and 2.22 mmol g^–1^), and C_2_H_6_ uptakes (4.44 and 3.36 mmol g^–1^) for **NPU-2** and **NPU-3** at 298 K and 100 kPa vs **NPU-1**, a similar phenomenon for C_2_H_2_ and C_2_H_6_ adsorption selectivity over C_2_H_4_ was observed. This trend also occurred in the 273 K C2 sorption isotherms. The C_2_H_2_ and C_2_H_6_ uptakes for **NPU-1** exceed the values of 4.55/3.79 mmol g^–1^ for **TJT-100** and 3.50/4.47 mmol g^–1^ for **Azole-Th-1**.

Adsorption selectivity is an important metric when evaluating separation performance. Ideal Adsorbed Solution Theory^51^ (IAST) was used to calculate the adsorption selectivity of C_2_H_2_/C_2_H_4_ and C_2_H_6_/C_2_H_4_ for equimolar gas mixtures at 298 K and 100 kPa, after fitting the single-component 298 K adsorption isotherms to the Langmuir–Freundlich equation (Supplementary Figures 16–18). The adsorption selectivity values for C_2_H_6_/C_2_H_4_ and C_2_H_2_/C_2_H_4_ were found to be 1.32 and 1.4 for **NPU-1**, 1.52 and 1.25 for **NPU-2**, and 3.21 and 1.32 for **NPU-3** (Figure 2g), making the **NPU-1/2/3** platform only the third reported example of C_2_H_2_ and C_2_H_6_ adsorption selectivity vs C_2_H_4_. The C_2_H_6_/C_2_H_4_ IAST selectivity values for **NPU-1/2/3** are comparable to those of the other C_2_H_6_-selective sorbents (1.95 for **MUF-15**,^52^ 1.8 for **IRMOF-8**,^53^ 1.76 for **NUM-7a**,^54^ and 1.6 for **JNU-2**(55)).

To evaluate the interaction strengths of C_2_H_2_, C_2_H_4_, and C_2_H_6_ with **NPU-1/2/3**, the adsorption enthalpies (*Q*_st_) were calculated using the Clausius–Clapeyron equation by fitting the C_2_H_2_, C_2_H_4_, and C_2_H_6_ adsorption isotherms at 273 and 298 K to the virial equation (detailed fitting curves are given in Supplementary Figures 22–24). As expected, **NPU-1/2/3** interact somewhat more strongly with C_2_H_2_ and C_2_H_6_ than with C_2_H_4_. Figure 2h presents C_2_H_2_ (27.88 kJ mol^–1^ for **NPU-1**, 20.98 kJ mol^–1^ for **NPU-2**, and 19.93 kJ mol^–1^ for **NPU-3**) and C_2_H_6_ (29.10 kJ mol^–1^ for **NPU-1**, 19.64 kJ mol^–1^ for **NPU-2**, and 18.71 kJ mol^–1^ for **NPU-3**) adsorption enthalpies relative to C_2_H_4_ (23.95 kJ mol^–1^ for **NPU-1**, 18.18 kJ mol^–1^ for **NPU-2**, and 17.79 kJ mol^–1^ for **NPU-3**) at low loadings. Considering the differences between the low C2 loading enthalpies, particularly those for C_2_H_2_ and C_2_H_6_, the decreasing *Q*_st_ trend holds across the full C2 loading range: *Q*_st_(C_2_H_2_) ≈ *Q*_st_(C_2_H_6_) > *Q*_st_(C_2_H_4_). Increased adsorption enthalpy for C2 hydrocarbons with decreasing pore size can be attributed to stronger binding in tighter pores.^11,56^ We note that a relatively low adsorption enthalpy value means a relatively low energy footprint for sorbent regeneration.

**Figure 3:**
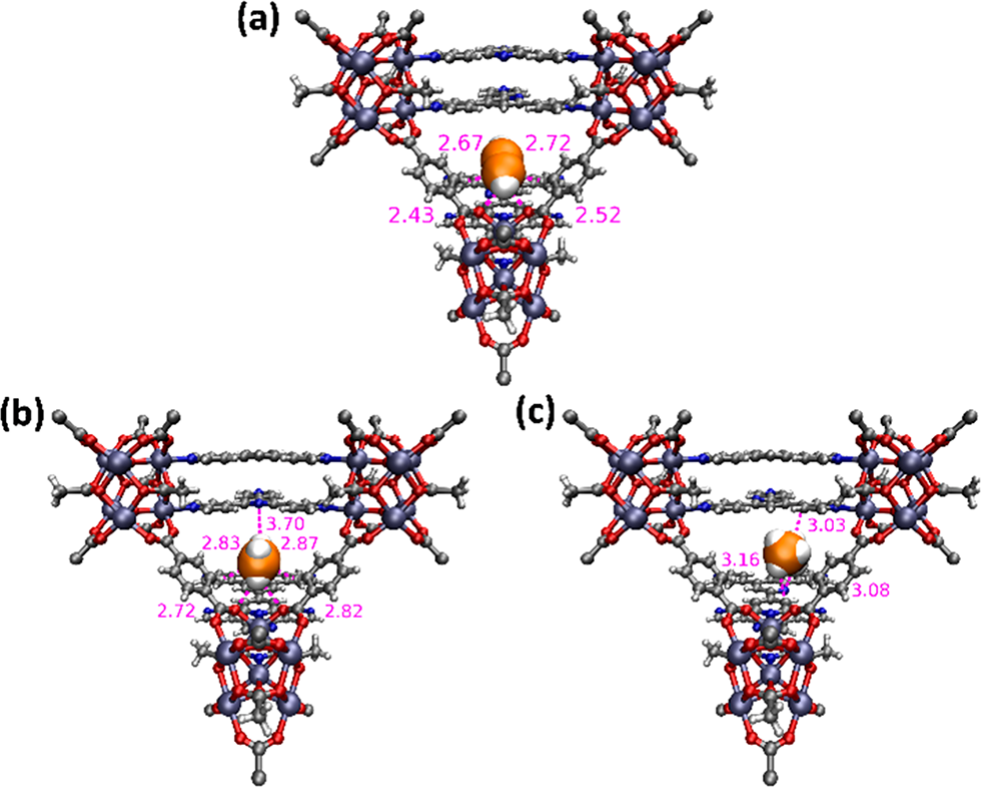
Molecular modeling determined primary adsorption sites of (a) C_2_H_2_, (b) C_2_H_4_, and (c) C_2_H_6_ in NPU-1. Adsorbed C2 molecules are presented in space-filling mode (C(MOM), gray; C(C2 gas), orange; H, white; O, red; N, blue; Mn, purple).

Molecular simulations were performed to gain insight into the nature of the binding sites for C_2_H_2_, C_2_H_4_, and C_2_H_6_ in **NPU-1** (see Supporting Information for full details). The modeling studies revealed that C_2_H_2_ localizes in a triangular pocket formed by two BDC linkers and one Tripp linker (Figure 3a). C_2_H_2_ molecules interact with electronegative carboxylate O atoms of the BDC linkers through their CH moieties. C_2_H_2_ molecules also interact with the aromatic rings of BDC and Tripp linkers. C_2_H_4_ was found to adsorb at the same location as C_2_H_2_, although its interaction distances are longer (Figure 3b), an indication that C_2_H_4_ is likely to exhibit weaker interactions with **NPU-1**. The calculated averaged classical potential energies for both adsorbates about this position (see Supporting Information for details) also support weaker interactions with C_2_H_4_. In essence, the *D*_2*h*_ symmetry of C_2_H_4_ offers a less favorable fit compared to the *D*_∞*h*_ symmetry of C_2_H_2_.

In contrast to C_2_H_2_ and C_2_H_4_, C_2_H_6_ is too bulky to be adsorbed in the triangular channels, and the modeling studies suggested that the most favorable binding site for C_2_H_6_ is between two adjacent Tripp linkers (Figure 3c). Its larger molecular dimensions and the presence of multiple H atoms allow C_2_H_6_ to engage in multiple weak interactions with surrounding Tripp linkers. C_2_H_6_ can also interact with the phenyl ring of a nearby BDC linker. This binding site is quite favorable, as the potential energy for C_2_H_6_ at this site is comparable to that for C_2_H_2_ at its energy minimum position on the basis of simulated annealing calculations (see Supplementary Table 3). Overall, molecular simulations support the following trend in sorbent–sorbate interactions for **NPU-1**: C_2_H_2_ ≈ C_2_H_6_ > C_2_H_4_. This trend is consistent with the experimental findings.

**Figure 4:**
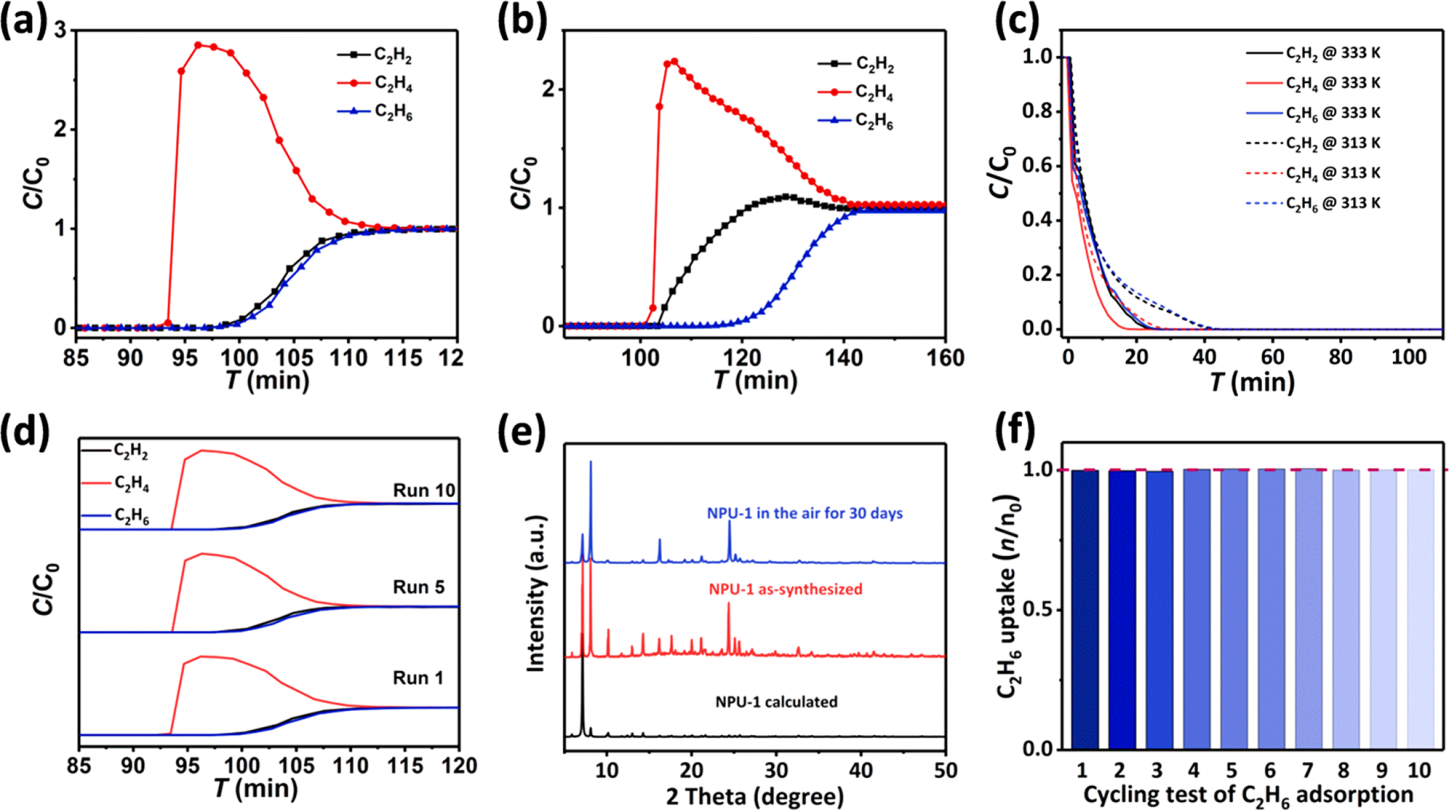
Dynamic breakthrough data and stability test. Experimental breakthrough curves at 298 K for C_2_H_2_/C_2_H_4_/C_2_H_6_ separation (1:1:1 mixture; total gas pressure of 100 kPa; total gas flow of 2.1 cm^3^ min^–1^) based on (a) **NPU-1** column with 2.9 g sample and (b) **NPU-2** column with 2.5 g sample. (c) Temperature-programmed desorption curves for **NPU-1**-packed column activated at 313 and 333 K under He flow of 20 cm^3^ min^–1^. (d) Dynamic breakthrough data of **NPU-1** in the 1st, 5th, and 10th cycles for ternary gas separation (*C*, outlet gas concentration; *C*_0_, inlet gas concentration). (e) PXRD patterns of **NPU-1** under different conditions. (f) Ten-cycle comparison of C_2_H_6_ adsorption capacity at 298 K and 100 kPa for **NPU-1** (*n*_0_, C_2_H_6_ uptake for first cycle; *n*, C_2_H_6_ uptake for the specific cycle).

To evaluate separation performance, dynamic column breakthrough experiments were performed for **NPU-1** and **NPU-2**. Two separate columns with tightly packed powder samples of 2.9 g of **NPU-1** and 2.5 g of **NPU-2** were prepared by pre-activating at 353 K under He flow (flow rate of 20 cm^3^ min^–1^). In a typical breakthrough experiment at 298 K, a 1:1:1 mixture of C_2_H_2_/C_2_H_4_/C_2_H_6_ at a total gas pressure of 100 kPa was passed through the packed adsorbent column, and the outlet gas signal was detected by gas chromatography. As shown in Figure 4a, **NPU-1** effectively captured C_2_H_2_ and C_2_H_6_ from the 1:1:1 gas mixture and afforded polymer-grade C_2_H_4_ in the effluent stream. Ethylene breakthrough occurred first at ca. 93 min, followed by C_2_H_2_ and C_2_H_6_ at ca. 100 min. Before the breakthrough of C_2_H_2_ and C_2_H_6_ from the column outlet, C_2_H_4_ with polymer-grade purity (>99.9%) was harvested, establishing the ability of **NPU-1** to produce polymer-grade ethylene from 1:1:1 C_2_H_2_/C_2_H_4_/C_2_H_6_ in a single separation step. Furthermore, dynamic breakthrough experiments with less **NPU-1** (1.3 g) exhibited similar separation performance but with slightly reduced working capacity (Supplementary Figure 31). For the **NPU-2** column, the breakthrough curve follows the same time sequence (C_2_H_4_ first, C_2_H_2_ second, and C_2_H_6_ last) as **NPU-1**, but ethylene in high purity was not afforded because of the very close breakthrough times of C_2_H_2_ and C_2_H_4_. This result was presumably a consequence of the lower adsorption enthalpy of all three gases for **NPU-2** vs **NPU-1** and the minimal differences between C_2_H_2_ and C_2_H_4_ isotherms in **NPU-2** at lower pressures. **NPU-3**, with even lower adsorption capacity and enthalpy, was not subjected to dynamic breakthrough experiments. To confirm the ease of regeneration of **NPU-1**, we conducted temperature-programmed desorption experiments after saturation of the separation column. As seen in Figure 4c, the **NPU-1** column can be fully activated after heating at 313 K for 40 min under He flow (10 cm^3^ min^–1^). Upon increasing the activation temperature to 333 K, the regeneration time was reduced to 25 min. To test the recycling ability of **NPU-1**, 10 cycles of ternary gas mixture breakthrough and single-gas ethane adsorption experiments were conducted. No performance loss was detected. In addition, the PXRD pattern of **NPU-1** collected after exposure to ambient air over a month verified its moisture stability.

## Conclusion

In summary, we report a new hexanuclear metal cluster, [Mn_6_(μ_3_-O)_2_(CH_3_COO)_3_]^6+^, that can serve as a node in three porous coordination networks (**NPU-1/2/3**) with the same **pacs** topology and high connectivity. Pore size and pore chemistry in the dual cages were controlled by linker ligand substitution, affording a pore environment suitable for effective separation of C_2_H_2_ and C_2_H_6_ from C_2_H_4_. **NPU-1** thereby enabled production of polymer-grade ethylene from a 1:1:1 C_2_H_2_/C_2_H_4_/C_2_H_6_ gas mixture at ambient conditions in one step. Sorbent–sorbate interactions, as delineated by molecular simulations, revealed that the dual cage nature of **NPU-1** is the reason for the observed performance.

## Supporting Information Available

Experimental details and characterization discussion, XRD patterns, sorption data, thermogravimetric analysis data, breakthrough experiment data of **NPU-1/2/3**, molecular simulations detail of **NPU-1**, and supplementary figures and tables (PDF)

## Accession Codes

CCDC 2032374–2032376 contain the supplementary crystallographic data for this paper. These data can be obtained free of charge via www.ccdc.cam.ac.uk/data_request/cif, or by emailing data_request@ccdc.cam.ac.uk, or by contacting The Cambridge Crystallographic Data Centre, 12 Union Road, Cambridge CB2 1EZ, UK; fax: +44 1223 336033.
