# Supplementary material for: Pore Engineering for One-Step Ethylene Purification from a Three-Component Hydrocarbon Mixture
Source: J Am Chem Soc. 2021 Jan 13;143(3):1485–92. doi: 10.1021/jacs.0c11247 (PMC8297724; doi:10.1021/jacs.0c11247)
Supplement: Supplementary file 1 — ja0c11247_si_001.pdf [file ja0c11247_si_001.pdf]

Supplementary Information

Pore engineering for one-step ethylene purification from a three-component hydrocarbon mixture

Baoyong Zhu,[2†] Jian-Wei Cao,[1†] Soumya Mukherjee,[3] Tony Pham,[4] Tao Zhang,[1] Teng Wang,[1] Xue Jiang,[1] Katherine A. Forrest,[4] Michael J. Zaworotko[3*] and Kai-Jie Chen[1*]

[1]Key Laboratory of Special Functional and Smart Polymer Materials of Ministry of Industry and Information Technology, Xi'an Key Laboratory of Functional Organic Porous Materials, School of Chemistry and Chemical Engineering, Northwestern Polytechnical University, Xi'an, Shaanxi 710072, P.R. China

[2]School of Chemistry and Chemical Engineering, Dezhou University, Dezhou, 253023, P.R. China.

[3]Bernal Institute, Department of Chemical Sciences, University of Limerick, Limerick V94 T9PX, Republic of Ireland.

[4]Department of Chemistry, University of South Florida, 4202 East Fowler Avenue, CHE205, Tampa, Florida 33620-5250, United States.

*E-mail: xtal@ul.ie; ckjiscon@nwpu.edu.cn

†These authors contributed equally in this work.

Synthesis and Methods

General

All reagents except 2,4,6-tris (4-pyridyl) pyridine (tripp, TPP) were commercially available and used as received without further purification. Tripp was based on previously reported literature.[1] Power X-Ray diffraction (PXRD) data was collected by Rigaku-Miniflex-600 diffractometer at a scanning rate of 5° min^{-1}. Cu K_α radiation was used for the diffraction experiments.TGA data was obtained using STA 449 F1 Jupiter Thermogravimetric Analyzer. The sample is heated from 25 °C to 850 °C under N_2 atmosphere at a heating rate of 5 °C min^{-1}. XPS data of Mn element was obtained using Kratos Axis Ultra DLD X-ray photoelectron spectroscopy. IR spectra data was collected by Nicolet IS5 infrared spectrometer (KBr pellet, 400-4000 cm^{-1}).

Synthesis of NPU-1, NPU-2 and NPU-3

For **NPU-1**, a mixture of $Mn(OAc)_2{\cdot}4H_2O$ (0.040 g), H_2BDC (0.016 g), Tripp (0.016 g), DMA (3 mL), HCOOH (0.2 mL) and HBF_4 (0.12 mL) was sealed in a 25 mL Teflon-lined stainless-steel reactor at 100 °C for 10 days, which was then slowly cooled to room temperature. After decanting the mother liquor, the light yellow crystal was rinsed three times with fresh DMA (5 mL × 3) and stored in DMA solvent. (73.5% yield based on metal content). **NPU-2** and **NPU-3** were prepared in a similar way as **NPU-1**, by using H_2NPDA (0.022 g) and H_2BPDC (0.024g) in place of H_2BDC, respectively. The yield of harvested light yellow crystals is *ca*. 55.9% and 56.9% based on metal content for **NPU-2** and **NPU-3**, respectively.

X-ray crystallography data based structural determination

Single crystals of **NPU-1**, **NPU-2** and **NPU-3** suitable for X-ray analysis were selected directly from the above synthesized samples. These crystals were mounted on glass fibers and then the crystallographic data collections were carried out on a Bruker Smart ApexII CCD area-detector diffractometer with graphite-monochromated Mo Kα radiation (λ = 0.71073 Å) at 23°C using ω and φ-scan technique. Data collection, frame integration, and data processing were performed with the use of the APEXII and SAINT program packages,[2] which was also used for the intensity corrections for the Lorentz and Polarization effects. Semiempirical absorption correction was applied based on the intensities of equivalent reflections with the use of the SADABS program.[3] The crystal structures were solved by direct method using SHELXT program[4] and refined

anisotropically for all non-hydrogen atoms by full-matrix least squares on all F^2 data using SHELXL.[5] The single suite WINGX was used as an integrated system for all the crystallographic programs.[6] All hydrogen atoms were added according to theoretical models, assigned isotropic displacement parameters and allowed to ride on their respective parent atoms, the $U_{iso}(H) = 1.2U_{eq}(C)$ or $1.5U_{eq}(C)$. A crystallographic 3-fold axis runs through the centre of the Tripp node and the disordered atoms of the central pyridine ring were therefore refined as 2/3 carbon and 1/3 nitrogen. The SQUEEZE program implemented in PLATON was used to calculate the solvents disordered area and remove their contribution to the overall intensities in **NPU-1**, **NPU-2** and **NPU-3**. Crystal data and a structure determination summary are listed in Supplementary table 1. The X-ray crystallographic coordinates for the structures reported in this study are deposited at the Cambridge Crystallographic Data Centre (CCDC) under deposition numbers 2032374 (**NPU-1**), 2032375 (**NPU-2**), 2032376 (**NPU-3**), and can be obtained free of charge (http://www.ccdc.cam.ac.uk/data_request/cif).

Single-component gas sorption experiment

The Micromeritics 3Flex instrument was used for collecting all the sorption isotherms. All the high-purity gases used in the single-gas adsorption experiments were purchased commercially: He (99.999%), CO_2 (99.995%), N_2 (99.999%), C_2H_2 (99.99%), C_2H_4 (99.999%), and C_2H_6 (99.999%). Before the single-gas sorption experiments, the sample was first fully exchanged with fresh CH_2Cl_2 three times daily for three days, followed by activation at 60 °C under high vacuum for 12 hours. When testing N_2 adsorption isotherms, the temperature was controlled at 77 K using a Dewar bottle containing 4 L of liquid N_2. The precise control of 273 and 298 K were implemented by the DC-2006 of Ningbo Scientz Biotechnology, which contains a cycle control system of ethylene glycol-H_2O mixture. The sample was degassed at 25 °C under high vacuum for 6 hours to regenerate at every interval of two independent isotherms.

Gas sorption enthalpy and selectivity calculation

Langmuir-Freundlich fit

The isotherm data for C_2H_6, C_2H_4 and C_2H_2 in **NPU-1/2/3** were fitted with the single-site Langmuir-Freundlich model.

$$q = Q_{sat} \frac{b_A p^v}{1 + b_A p^v}$$

b_A is Langmuir-Freundlich constant for species i at adsorption site A (Pa^{-v}). Q_{sat} is saturation loading (mol kg^{-1}). q_i component molar loading of species i (mol kg^{-1}). p_i is partial pressure of species i in mixture (Pa). v is Freundlich exponent (dimensionless).

IAST selectivity calculation

Adsorption selectivity of C_2H_2/C_2H_4, C_2H_6/C_2H_4, mixed gases was predicted from single component adsorption isotherms using Ideal Adsorbed Solution Theory (IAST).[7]

$$S = \frac{X_A/X_B}{Y_A/Y_B}$$

Where S is the selectivity of component A relative to B. X_A and X_B are the molar fractions of components A and B in the adsorption phase, respectively. Y_A and Y_B are molar fractions of components A and B in the gas phase, respectively.

Absorption enthalpy calculation

Using data from 273 and 298 K, the adsorption enthalpy was calculated fitting by the Virial equation. P is the pressure described in Pa, N is the adsorbed amount in mmol/g, T is the temperature in K, a_i and b_i are Virial coefficients, and m and n are the number of coefficients used to describe the isotherms. Q_{st} is the coverage-dependent enthalpy of adsorption and R is the universal gas constant.

$$\mathrm{Ln\ P} = \mathrm{Ln\ N} + \sum_{i=0}^{m} a_i N^i + \sum_{i=0}^{n} \binom{n}{k} b_i N^i$$

$$Q_{st} = -R \sum_{i=0}^{m} a_i N^i$$

Dynamic gas breakthrough experiment

The breakthrough curves were recorded on a homemade apparatus. An equimolar gas mixture of C_2H_6, C_2H_4 and C_2H_2 (total gas pressure and flow: 100 kPa and 2.1 cm^3 min^{-1}) passes through the packing column filled with 2.9 g **NPU-1** or 2.5 g **NPU-2** at 298 K, and the outlet gas concentration was monitored by a gas chromatography analyzer (TCD-Thermal Conductivity Detector, detection

limit 0.1 ppm). Temperature programmed desorption data of the aboved mentioned **NPU-1** (2.9 g) column in Figure 5 was collected under a helium flow of 20 cm^3 min^{-1} at 40 or 60 °C, just after gas adsorption saturation of separation column reached. During gas breakthrough cycling tests, the sample in the column was regenerated under He flow of 20 cm^3 min^{-1} at 60 °C for an hour, after each breakthrough experiment.

Modeling Study

The binding sites for C_2H_2, C_2H_4, and C_2H_6 in **NPU-1** were determined through classical molecular simulations. All parametrizations and simulations were performed on the single X-ray crystallographic structure published herein for the material.

All atoms of **NPU-1** were treated with Lennard-Jones (LJ) parameters (ε and σ), point partial charges, and point polarizabilities in order to model repulsion/dispersion, stationary electrostatic, and many-body polarization interactions, respectively. The LJ parameters for all C and H atoms as well as the N atom of the central aromatic ring of the Tripp linker were taken from the Optimized Potentials For Liquid Simulations-All Atom (OPLS-AA) force field,[8] while those for the Mn, O, and metal-coordinated N atoms were taken from the Universal Force Field (UFF).[9] The partial charges for the chemically distinct atoms in **NPU-1** (Supplementary Figure 28) were determined through electronic structural calculations on different gas phase fragments that were selected from the crystal structure of the MOM. These calculations were performed using the NWChem *ab initio* software[10] with the 6-31G* basis set assigned to C, H, N, and O and the LANL2DZ ECP basis set[11-13] assigned to Mn. The exponential damping-type polarizability values for all C, H, N, and O atoms were taken from a carefully parametrized set provided by the work of van Duijnen and Swart.[14] The polarizability parameter for Mn^{2+} was calculated in previous work[15] and used herein. The simulation parameters for all chemically distinct atoms in **NPU-1** are provided in Supplementary **Table 2**.

Simulated annealing (SA) calculations[16] were performed for a single molecule of each adsorbate through a canonical Monte Carlo (*NVT*) process in a 2×2×1 supercell of **NPU-1**. All MOM atoms were kept fixed at their crystallographic positions throughout the simulations. A spherical cut-off distance corresponding to half the shortest supercell dimension length was used for the simulations. C_2H_2, C_2H_4, and C_2H_6 were modeled using polarizable potentials of the respective adsorbates that were developed previously.[17] The total potential energy of the MOM–adsorbate

system was calculated through the sum of the repulsion/dispersion, stationary electrostatic, and many-body polarization energies. These were calculated using the LJ potential,[18] the Ewald summation technique,[19, 20] and a Thole-Applequist type model,[21-24] respectively. SA calculations for each adsorbate utilized an initial temperature of 500 K, and this temperature was scaled by a factor of 0.99999 after every 10^3 Monte Carlo (MC) steps. The simulations continued until 10^6 MC steps were reached; at this point, the temperature of the system is below 10 K. All simulations were carried out using the Massively Parallel Monte Carlo (MPMC) code.[25, 26]

Supplementary Figure 29 shows the total potential energies plotted as a function of MC steps from the SA calculations for each adsorbate in **NPU-1**. The point at which the energies fluctuate around a constant minimum value is indicative of the adsorbate settling into a global minimum in the material. It can be observed that the energies associated with C_2H_2 localized at its energy minimum position are the lowest of the three adsorbates, which suggests that its interaction with the MOM is the most favorable. The binding of C_2H_6 at its energy minimum position corresponds to the second lowest energies, indicating that it displays slightly weaker interactions with the material than C_2H_2. When C_2H_4 settled into a global minimum in the MOM, the calculated energies were observed to be the highest. This implies that C_2H_4 exhibits the weakest interaction with the material of the three adsorbates considered. The *averaged* classical potential energies for C_2H_2, C_2H_4, and C_2H_6 localized about their energy minimum position in **NPU-1** are presented in **Supplementary Table 3**.

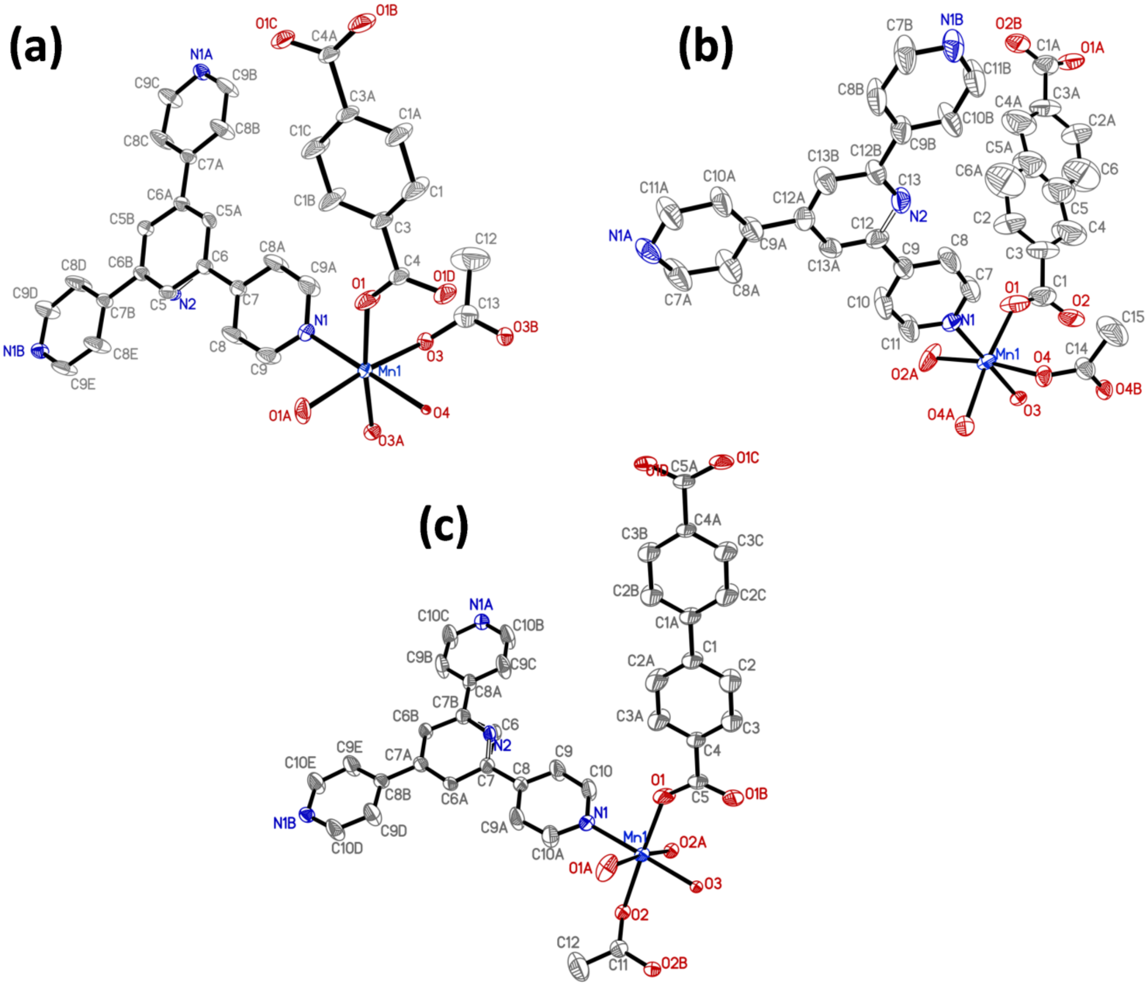

Supplementary Figure 1 | Coordination environments of the Mn centers with ellipsoids drawn at the 30% probability level in **NPU-1** (a), **NPU-2** (b), and **NPU-3** (c). Hydrogen atoms are omitted for clarity.

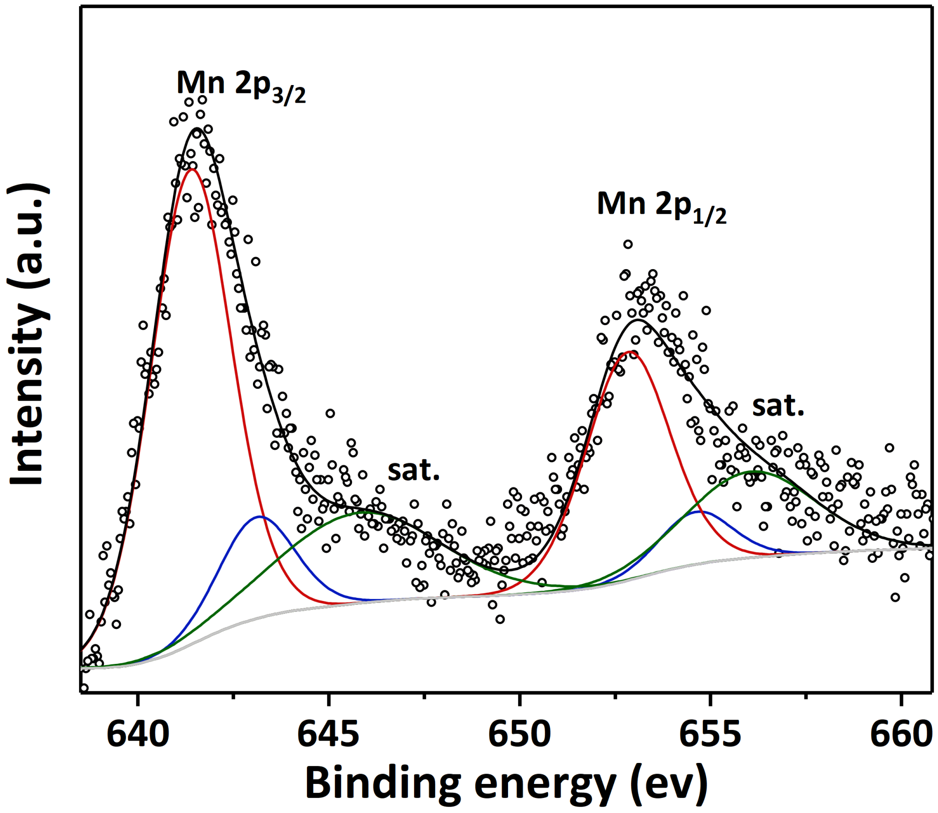

Supplementary Figure 2 | HRXPS spectra of Mn elements in **NPU-1**. The peak of the sample became very strong at 641.4 and 652.9 ev confirming the existence of Mn^{II}, while the peak at 643.2 and 654.7 ev confirmed the existence of Mn^{III}.

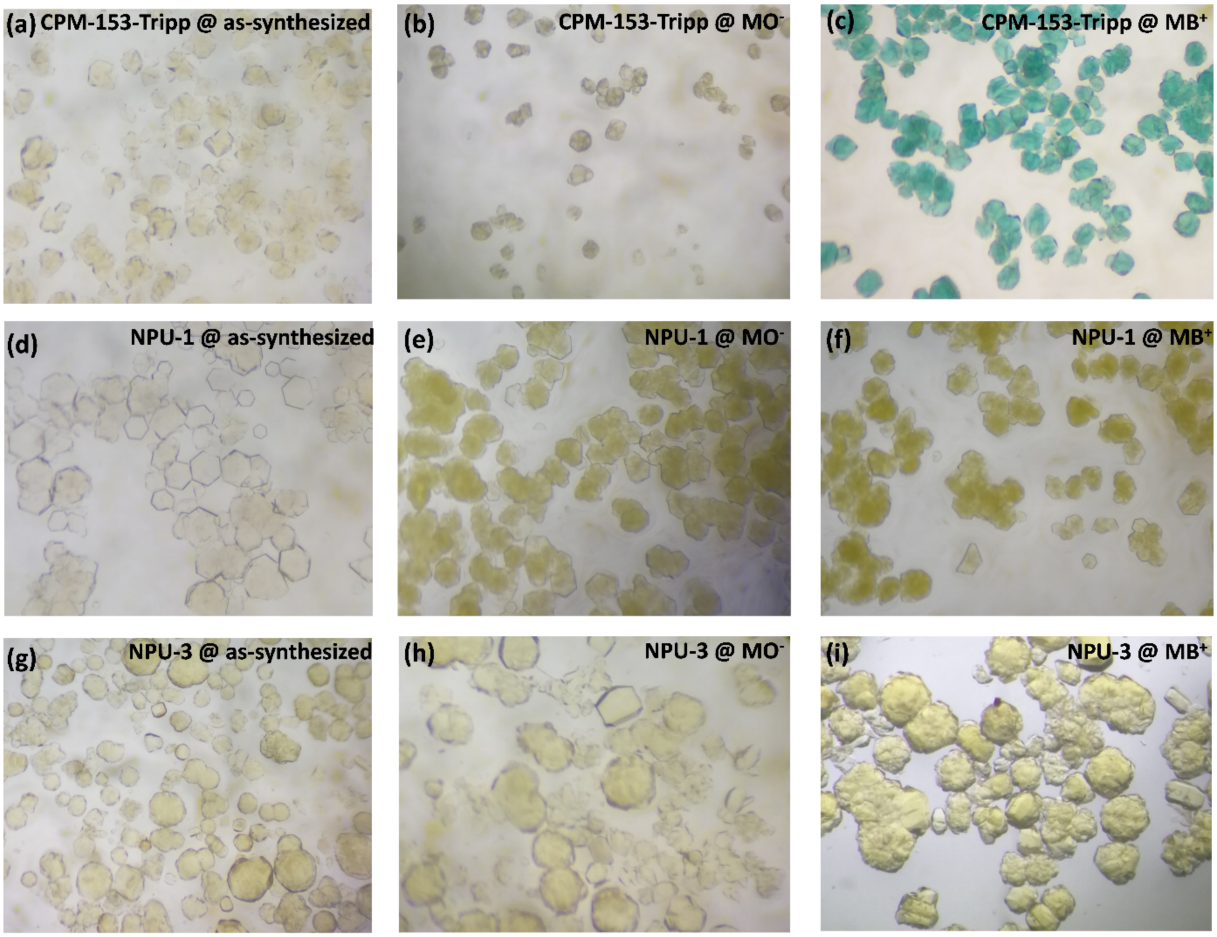

Supplementary Figure 3 | Optical microscope images of additional ion exchange experiments with the DMF solution of methyl orange (MO^-) and methylene blue (MB^+) for **CPM-153-Tripp** (a, b, c)**, NPU-1** (d, e, f) and **NPU-3** (g, h, i). (a) **CPM-153-Tripp** crystals as-synthesized. (b) **CPM-153-Tripp** crystals soaked in MO^- solution for four days. (c) **CPM-153-Tripp** crystals soaked in MB^+ solution for four days. (d) **NPU-1** crystals as-synthesized. (e) **NPU-1** crystals soaked in MO^- solution for four days. (f) **NPU-1** crystals soaked in MB^+ solution for four days. (g) **NPU-3** crystals as-synthesized. (h) **NPU-3** crystals soaked in MO^- solution for four days. (i) **NPU-3** crystals soaked in MB^+ solution for four days. **CPM-153-Tripp** with organic ligand of Tripp was synthesized based on a previously reported porous coordination network of **CPM-153** with organic ligand of TPT. [27] **CPM-153-Tripp** has very similar pore size and window size with **NPU-1**, and the successful ion exchange experiment of charged **CPM-153-Tripp** also indicate an absence of extra-framework counterions in **NPU-1.**

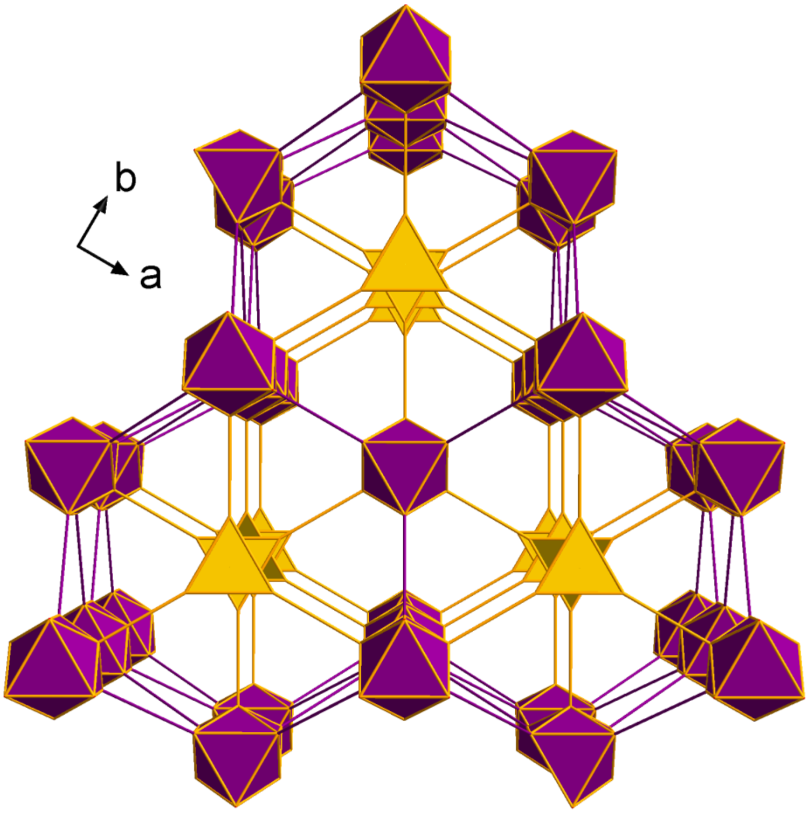

Supplementary Figure 4 | ** Topology of **NPU-1/2/3. Three-dimensional structures of **NPU-1/2/3** exhibit the binodal 3,9-connected network with **pacs** topology.

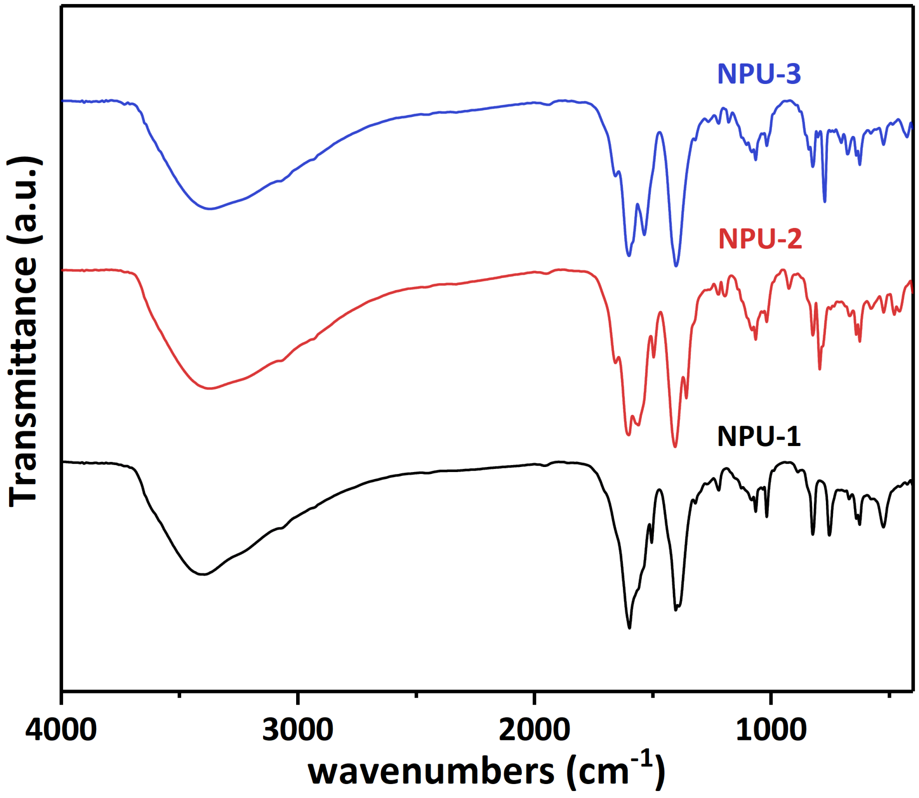

Supplementary Figure 5 | IR spectra of **NPU-1** (black), **NPU-2** (red) and **NPU-3** (blue).

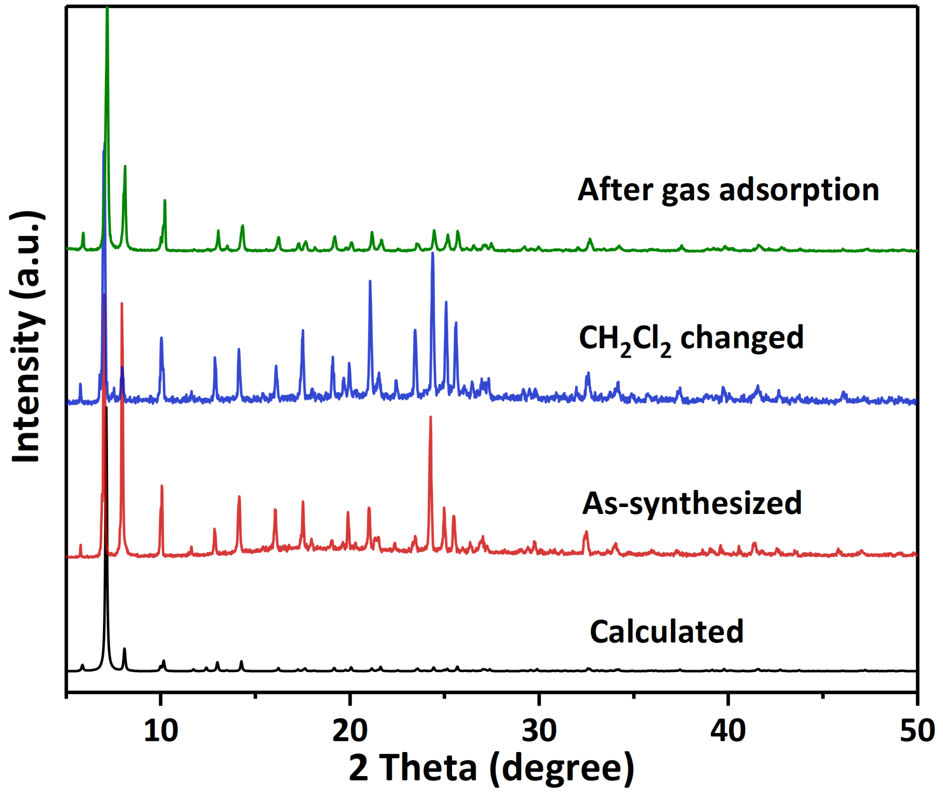

Supplementary Figure 6 | The PXRD patterns of **NPU-1**. The experimental results of as-synthesized (red), CH_2Cl_2 changed (blue) **NPU-1** and samples after gas adsorption (green).

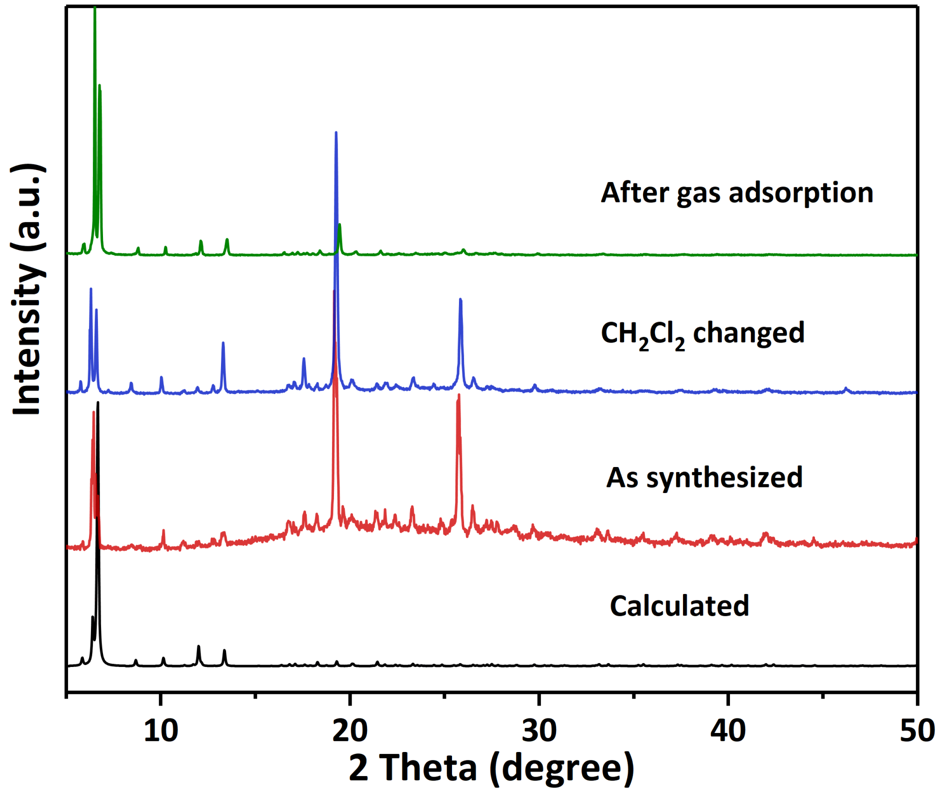

Supplementary Figure 7 | The PXRD patterns of **NPU-2**. The experimental results of as-synthesized (red), CH_2Cl_2 changed (blue) **NPU-2** and samples after gas adsorption (green).

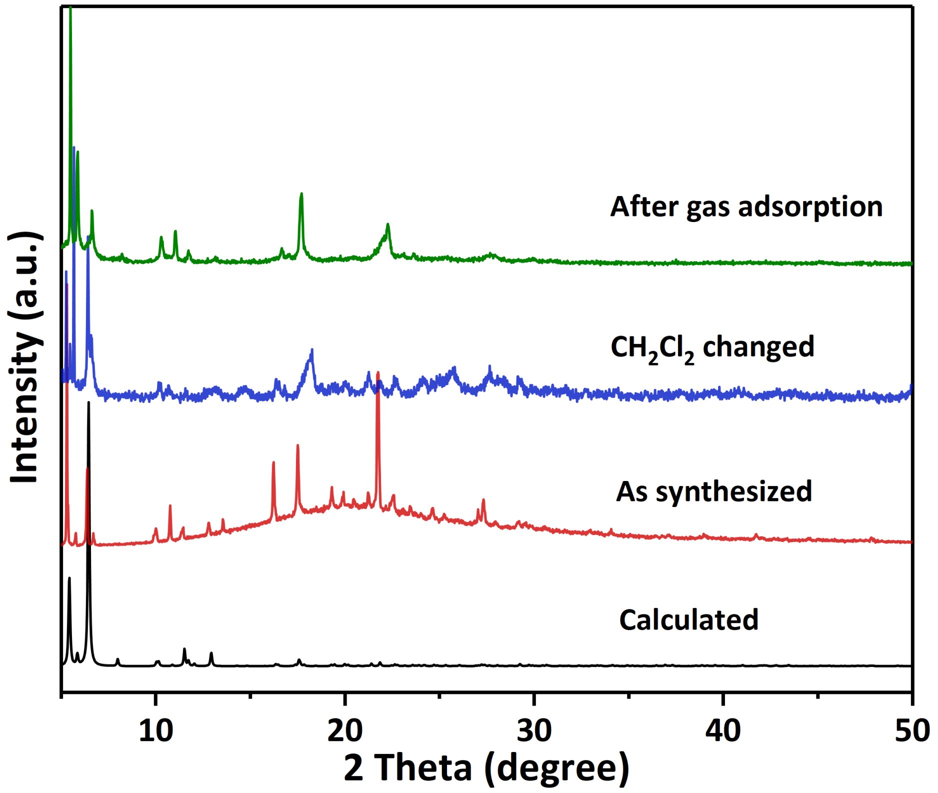

Supplementary Figure 8 | The PXRD patterns of **NPU-3**. The experimental results of as-synthesized (red), CH_2Cl_2 changed (blue) **NPU-3** and samples after gas adsorption (green).

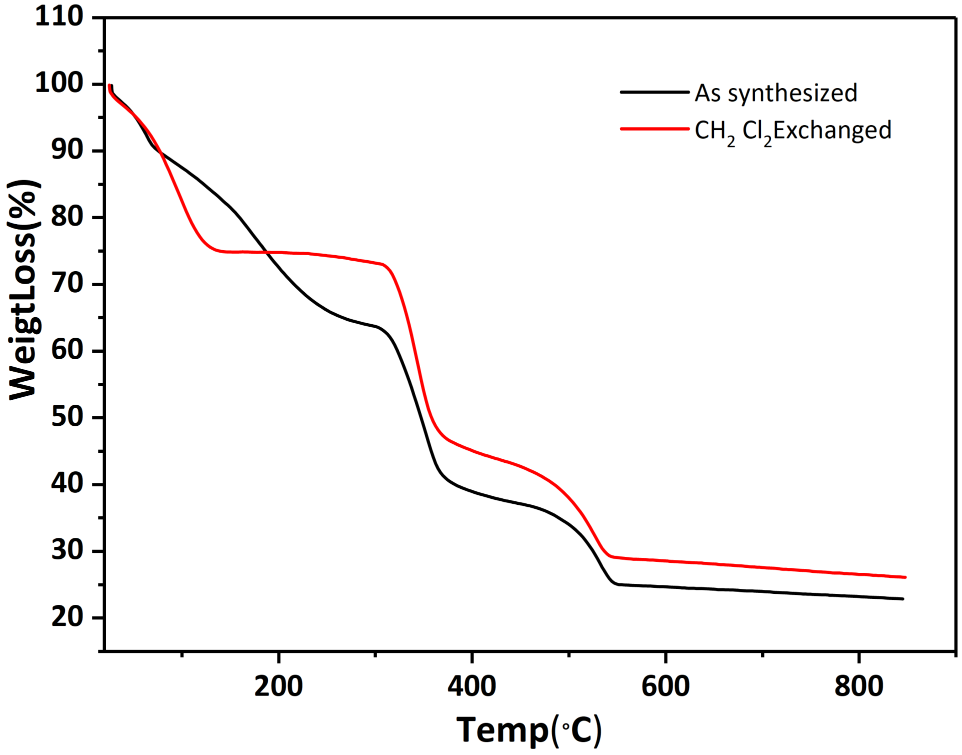

Supplementary Figure 9 | The TG analysis of NPU-1. The as-synthesized NPU-1 samples, and the samples soaked in CH_2Cl_2 for three days under N_2 atmosphere at a heating rate of 5°C per minute.

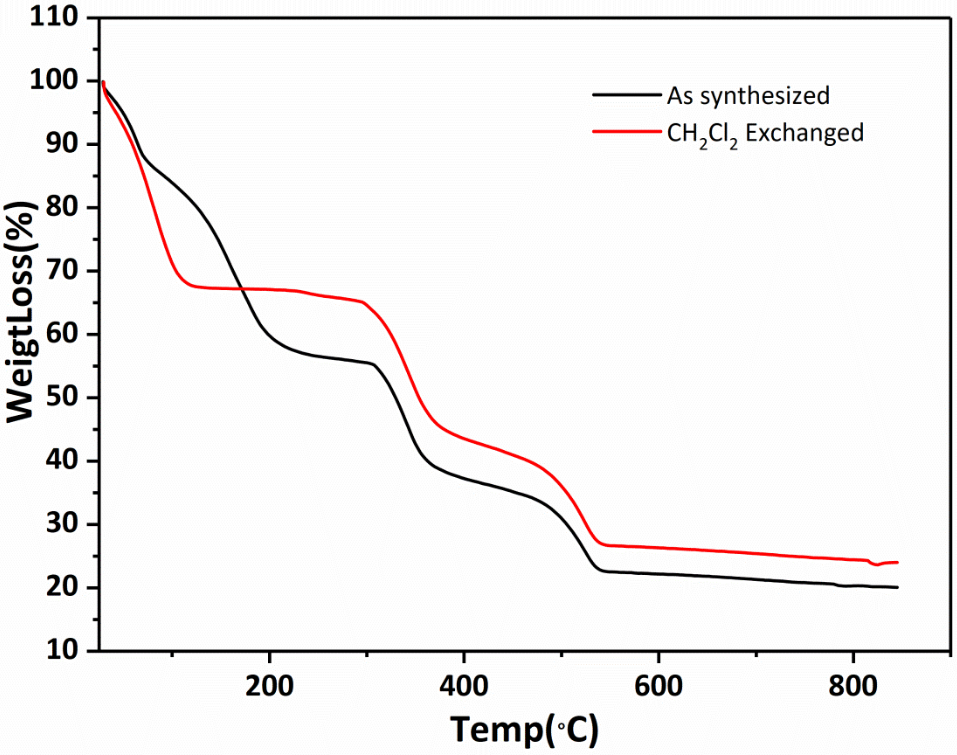

Supplementary Figure 10 | The TG analysis of NPU-2. The as-synthesized NPU-2 samples, and the samples soaked in CH_2Cl_2 for three days under N_2 atmosphere at a heating rate of 5°C per minute.

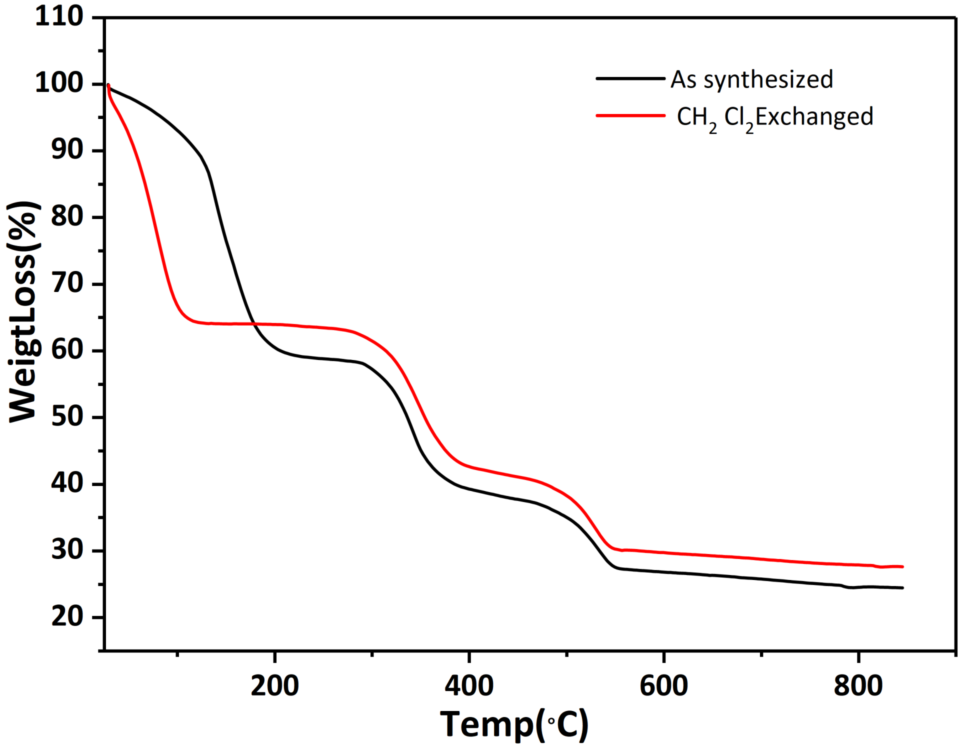

Supplementary Figure 11 | The TG analysis of NPU-3. The as-synthesized NPU-3 samples, and the samples soaked in CH_2Cl_2 for three days under N_2 atmosphere at a heating rate of 5°C per minute.

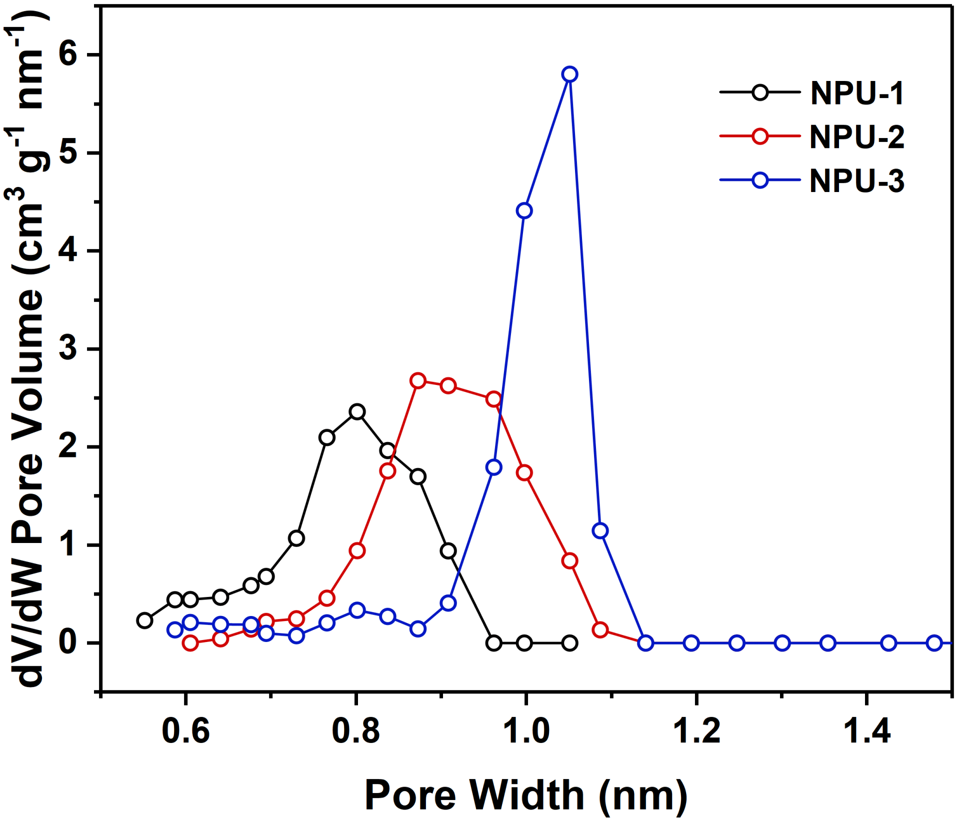

Supplementary Figure 12 | NLDFT model-based pore-size distribution data from 77 K N_2 sorption isotherms.

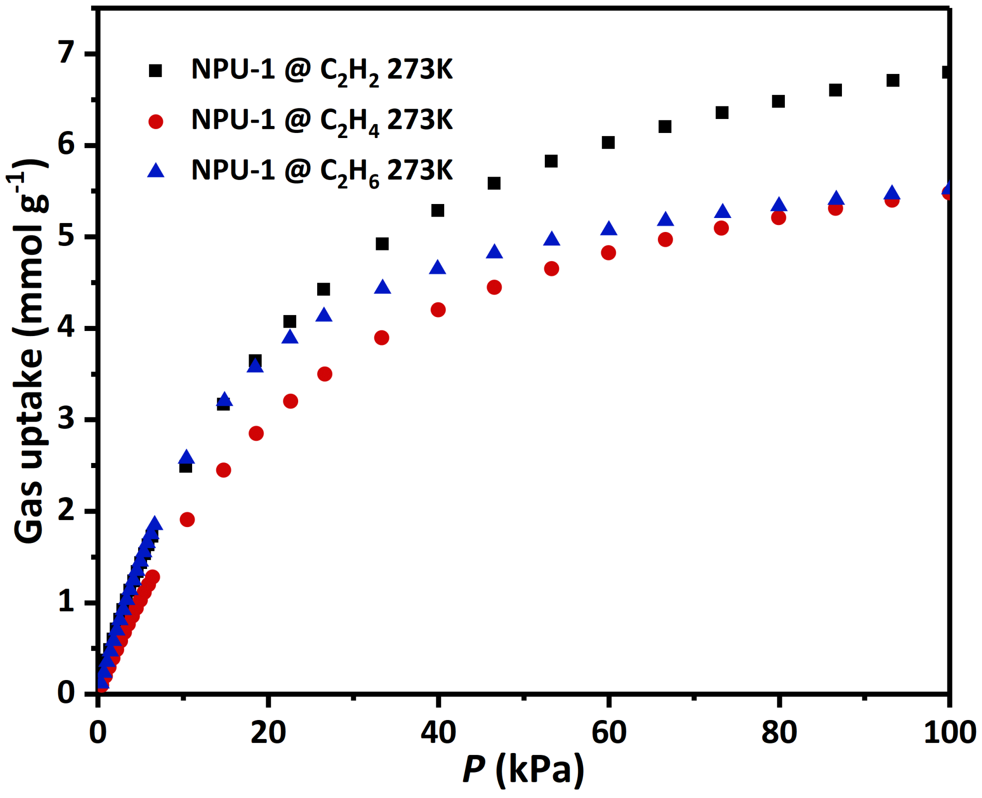

Supplementary Figure 13 | C_2H_2 (black square), C_2H_4 (red circle) and C_2H_6 (blue triangle) adsorption isotherms for activated **NPU-1** at 273 K.

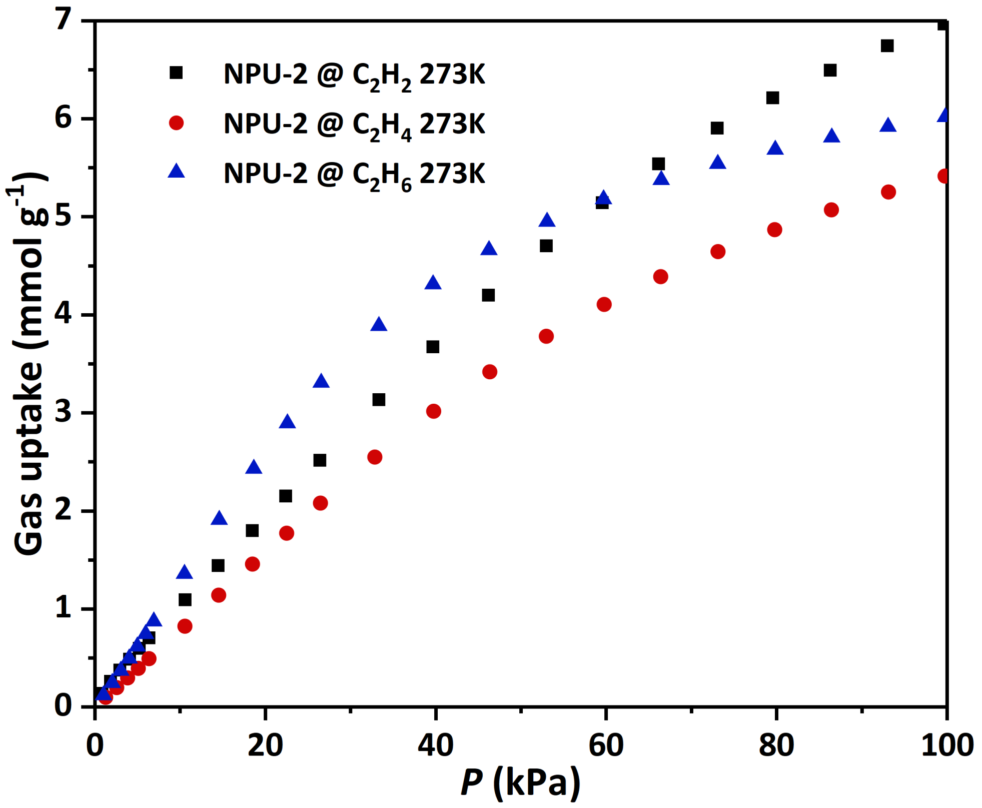

Supplementary Figure 14 | C_2H_2 (black square), C_2H_4 (red circle) and C_2H_6 (blue triangle) adsorption isotherms for activated **NPU-2** at 273 K.

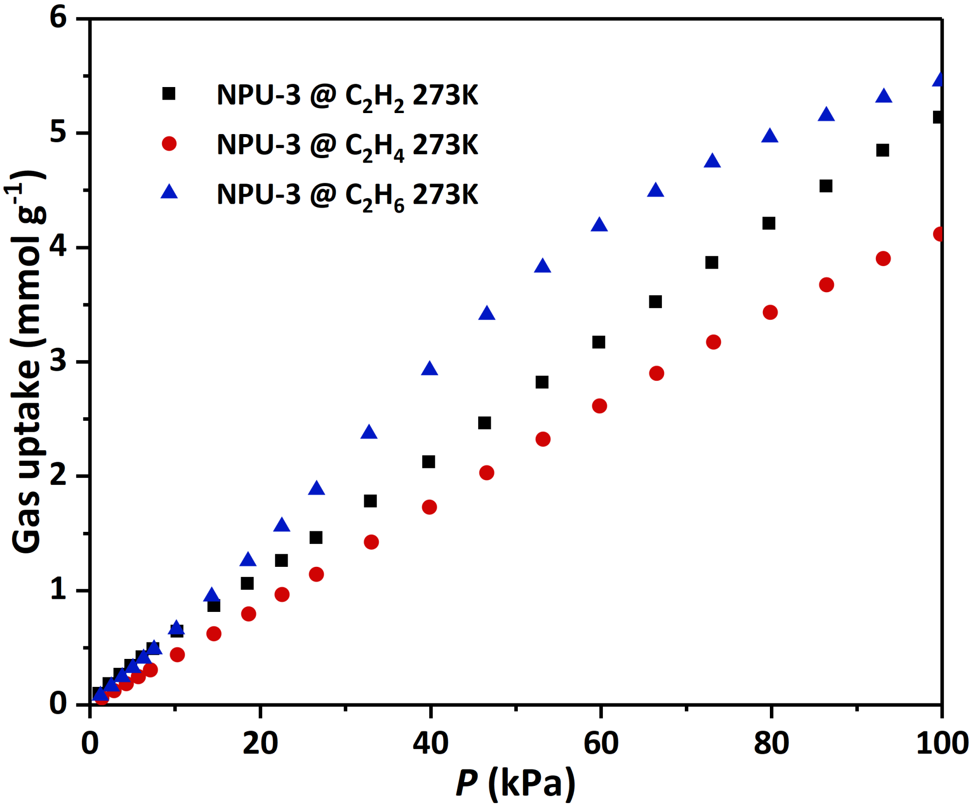

Supplementary Figure 15 | C_2H_2 (black square), C_2H_4 (red circle) and C_2H_6 (blue triangle) adsorption isotherms for activated **NPU-3** at 273 K.

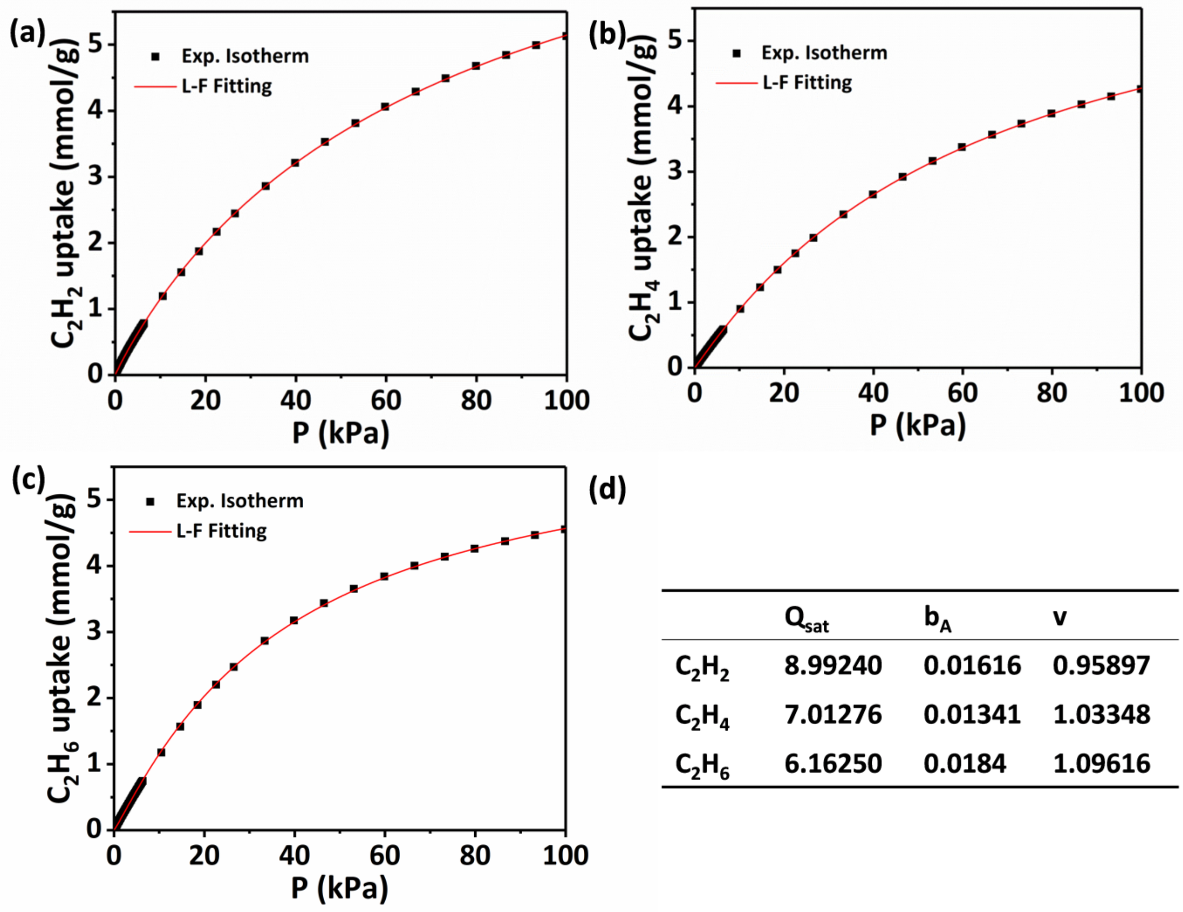

	Q_{sat}	b_A	v
C_2H_2	8.99240	0.01616	0.95897
C_2H_4	7.01276	0.01341	1.03348
C_2H_6	6.16250	0.0184	1.09616

Supplementary Figure 16 | Langmuir-Freundlich (L-F) fitting of C_2H_2 (a), C_2H_4 (b) and C_2H_6 (c) sorption data and fitting parameters (d) at 298K for **NPU-1**.

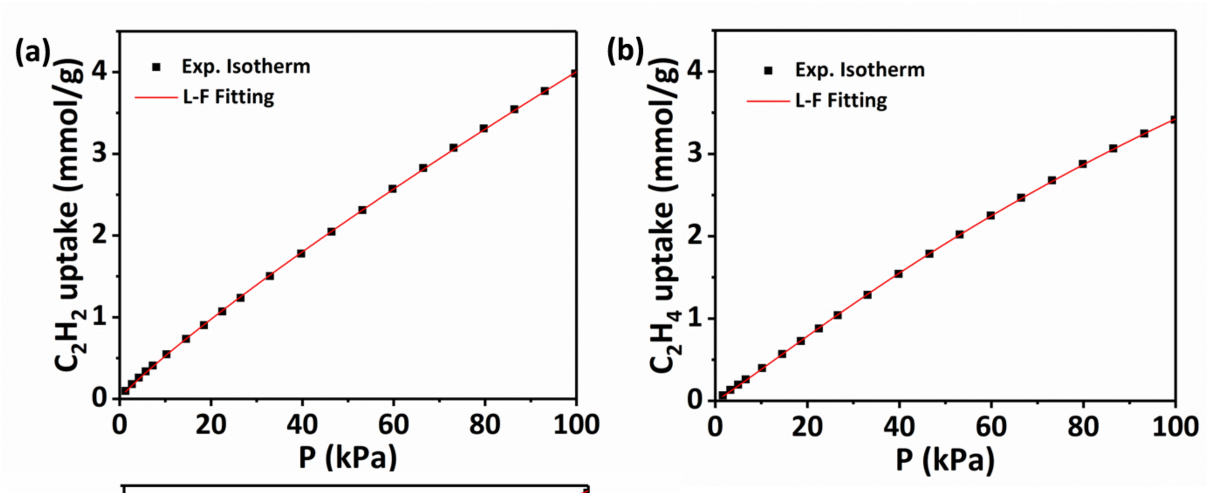

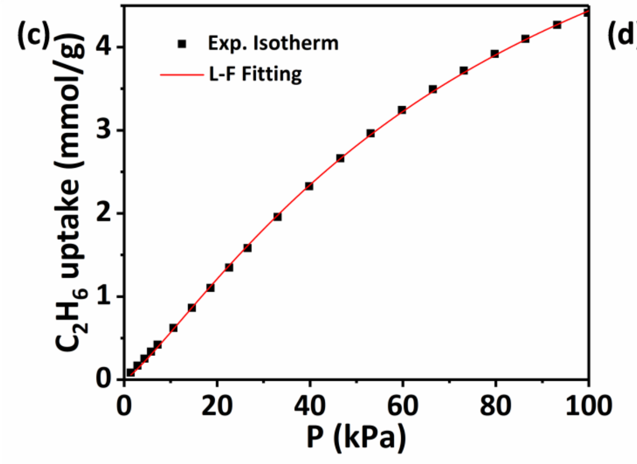

(d)

	Q_{sat}	b_A	v
C_2H_2	104.72938	6.40032E-4	0.89668
C_2H_4	11.52763	0.00278	1.09115
C_2H_6	7.79883	1.78009E-4	1.22519

Supplementary Figure 17 | Langmuir-Freundlich (L-F) fitting of C_2H_2 (a), C_2H_4 (b) and C_2H_6 (c) sorption data and fitting parameters (d) at 298K for **NPU-2**.

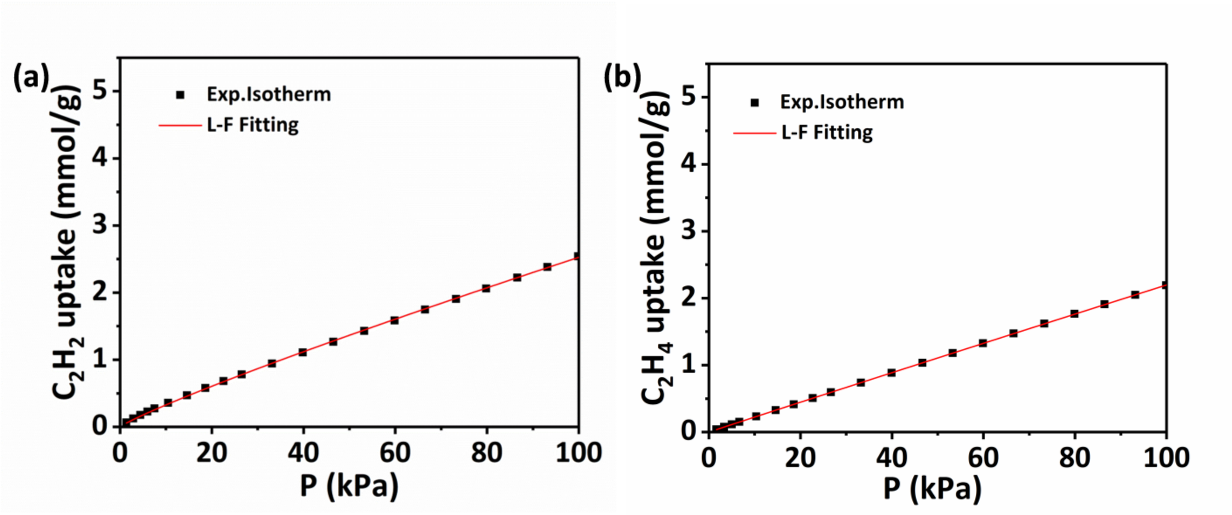

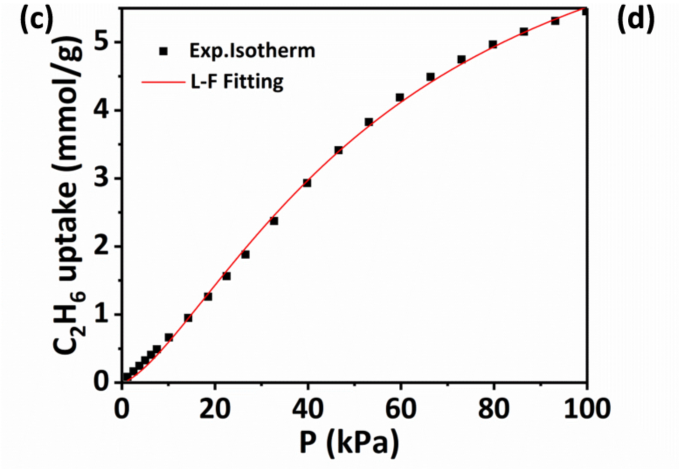

(d)

	Q_{sat}	b_A	v
C_2H_2	2707.09	1.56053E-5	0.88859
C_2H_4	346.115	6.57239E-5	0.99388
C_2H_6	8.00846	3.5601E-4	1.4431

Supplementary Figure 18 | Langmuir-Freundlich (L-F) fitting of C_2H_2 (a), C_2H_4 (b) and C_2H_6 (c) sorption data and fitting parameters (d) at 298K for **NPU-3**.

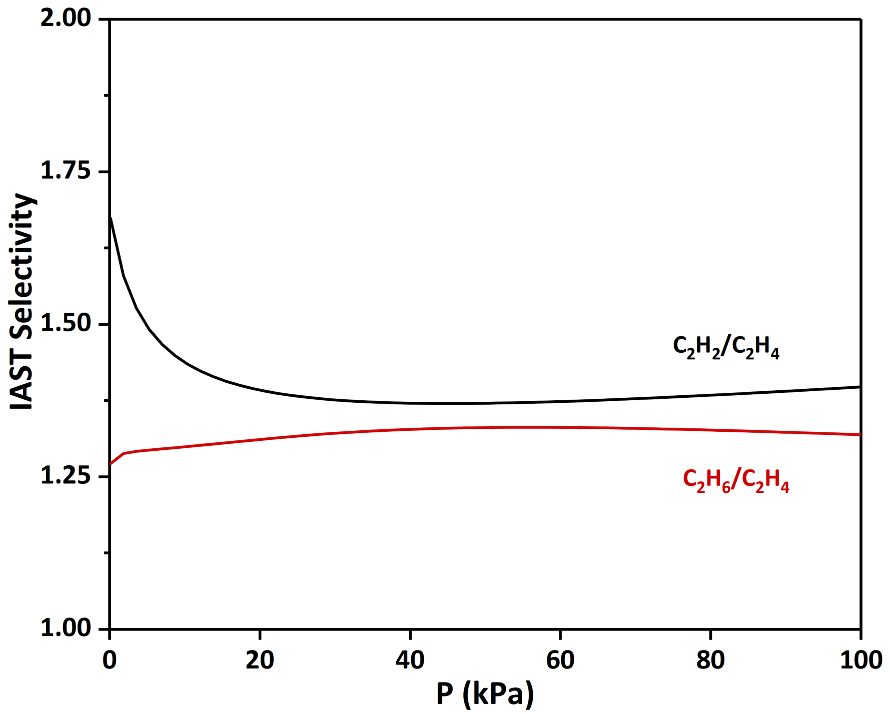

Supplementary Figure 19 | Calculated IAST selectivity of **NPU-1** for C_2H_2/C_2H_4 and C_2H_6/C_2H_4 with 1/1 ratio at 298K.

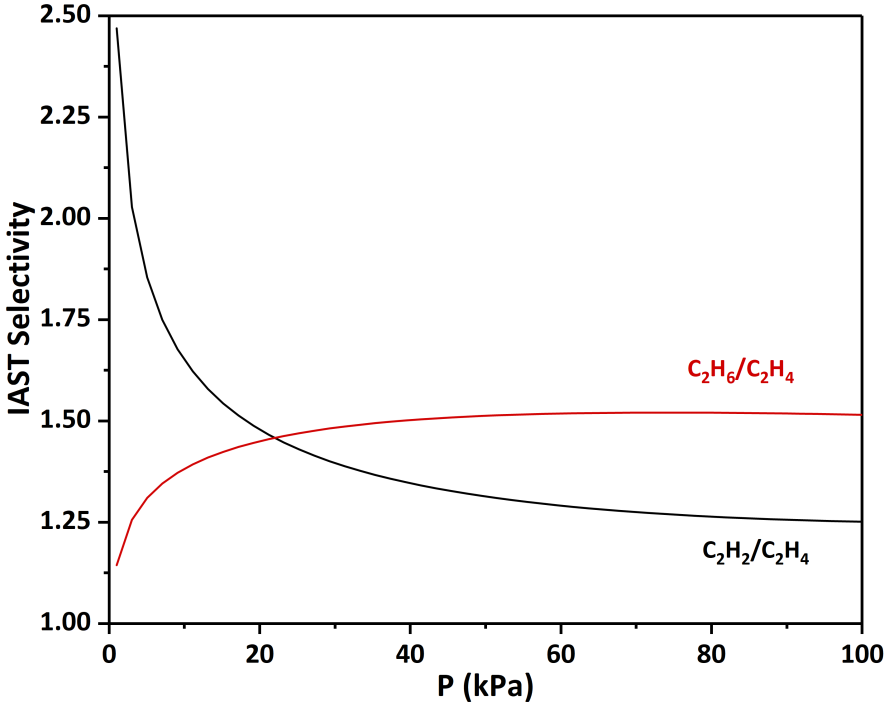

Supplementary Figure 20 | Calculated IAST selectivity of **NPU-2** for C_2H_2/C_2H_4 and C_2H_6/C_2H_4 with 1/1 ratio at 298K.

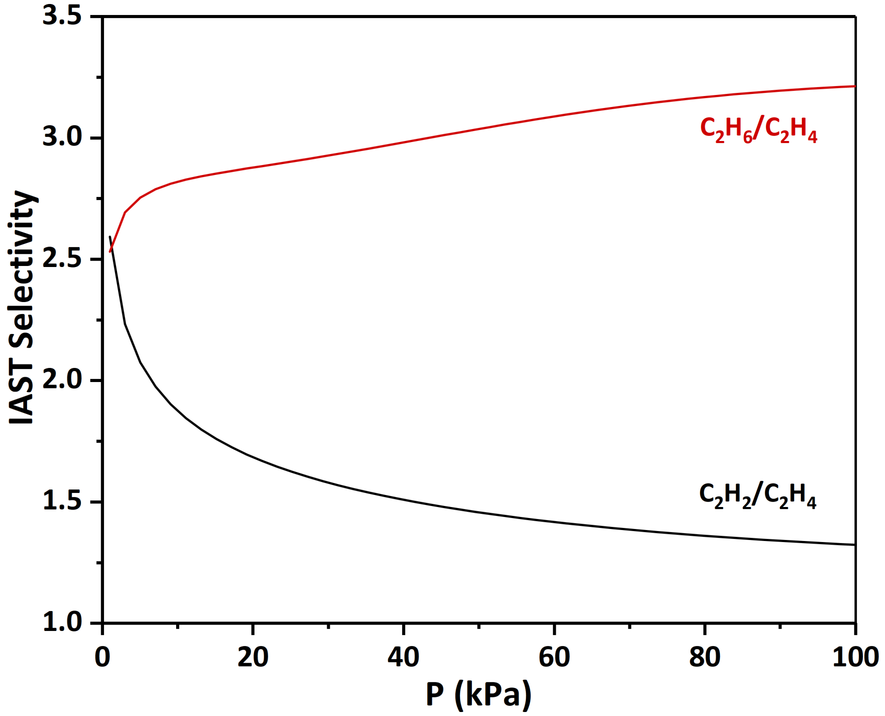

Supplementary Figure 21 | Calculated IAST selectivity of **NPU-3** for C_2H_2/C_2H_4 and C_2H_6/C_2H_4 with 1/1 ratio at 298K.

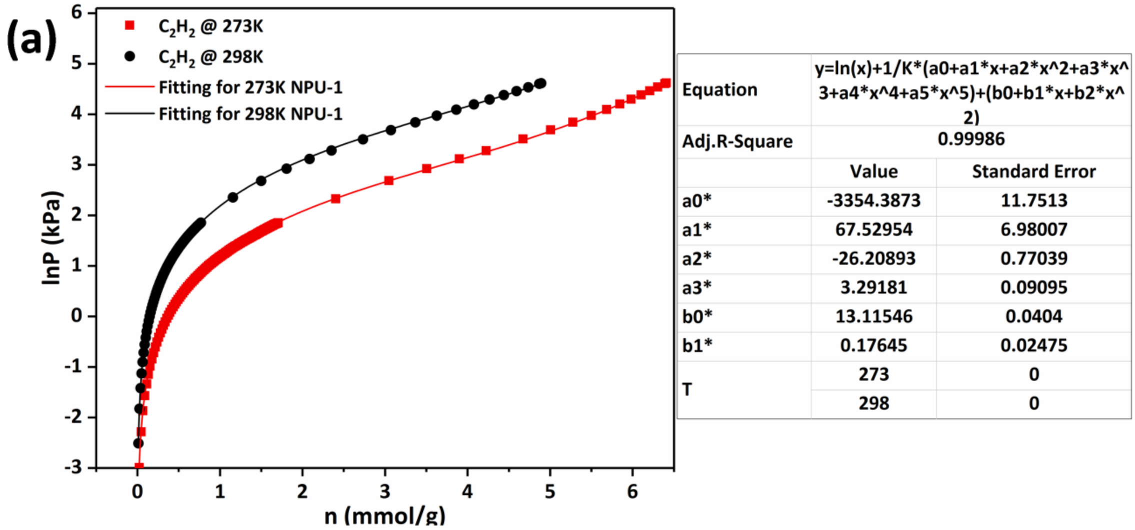

Equation	y=ln(x)+1/K*(a0+a1*x+a2*x^2+a3*x^3+a4*x^4+a5*x^5)+(b0+b1*x+b2*x^2)	
Adj.R-Square	0.99986	
	Value	Standard Error
a0*	-3354.3873	11.7513
a1*	67.52954	6.98007
a2*	-26.20893	0.77039
a3*	3.29181	0.09095
b0*	13.11546	0.0404
b1*	0.17645	0.02475
T	273	0
	298	0

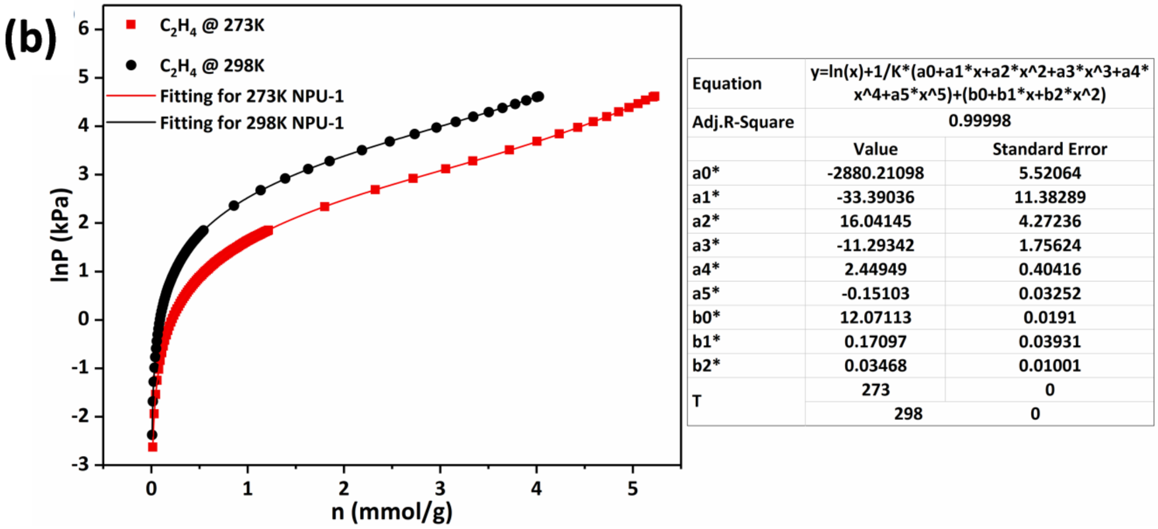

Equation	y=ln(x)+1/K*(a0+a1*x+a2*x^2+a3*x^3+a4*x^4+a5*x^5)+(b0+b1*x+b2*x^2)	
Adj.R-Square	0.99998	
	Value	Standard Error
a0*	-2880.21098	5.52064
a1*	-33.39036	11.38289
a2*	16.04145	4.27236
a3*	-11.29342	1.75624
a4*	2.44949	0.40416
a5*	-0.15103	0.03252
b0*	12.07113	0.0191
b1*	0.17097	0.03931
b2*	0.03468	0.01001
T	273	0
	298	0

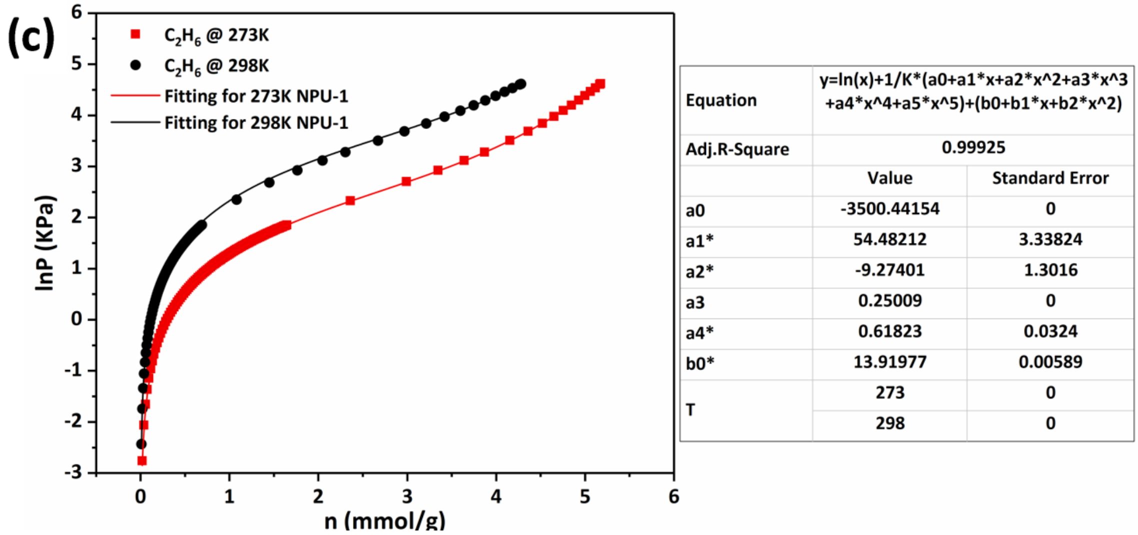

Equation	y=ln(x)+1/K*(a0+a1*x+a2*x^2+a3*x^3+a4*x^4+a5*x^5)+(b0+b1*x+b2*x^2)	
Adj.R-Square	0.99925	
	Value	Standard Error
a0	-3500.44154	0
a1*	54.48212	3.33824
a2*	-9.27401	1.3016
a3	0.25009	0
a4*	0.61823	0.0324
b0*	13.91977	0.00589
T	273	0
	298	0

Supplementary Figure 22 | Virial fitting of C_2H_2 (a), C_2H_4 (b) and C_2H_6 (c) adsorption data for **NPU-1** at 273 and 298 K.

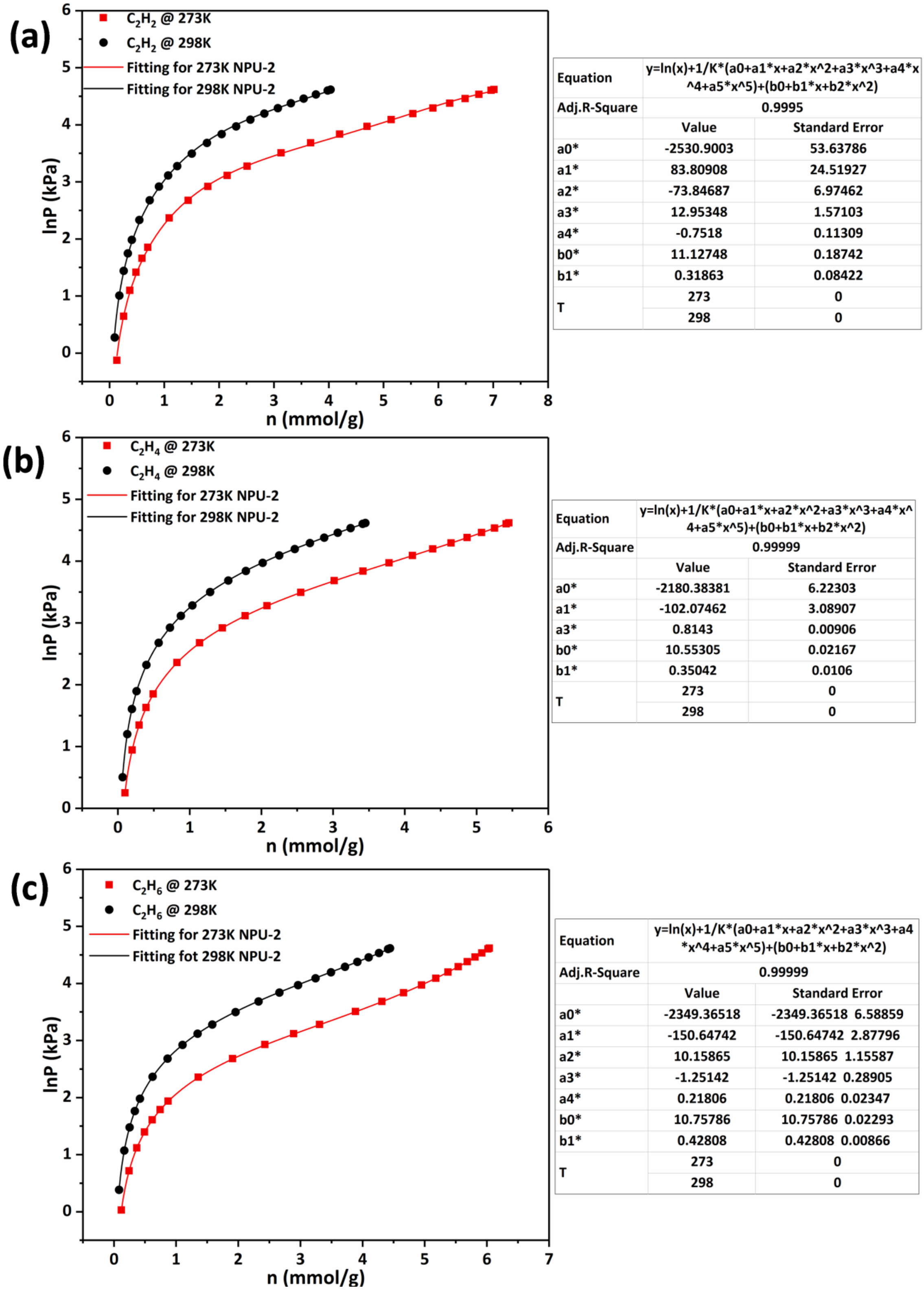

(a)

Equation	y=ln(x)+1/K*(a0+a1*x+a2*x^2+a3*x^3+a4*x^4+a5*x^5)+(b0+b1*x+b2*x^2)	
Adj.R-Square	0.9995	
	Value	Standard Error
a0*	-2530.9003	53.63786
a1*	83.80908	24.51927
a2*	-73.84687	6.97462
a3*	12.95348	1.57103
a4*	-0.7518	0.11309
b0*	11.12748	0.18742
b1*	0.31863	0.08422
T	273	0
	298	0

(b)

Equation	y=ln(x)+1/K*(a0+a1*x+a2*x^2+a3*x^3+a4*x^4+a5*x^5)+(b0+b1*x+b2*x^2)	
Adj.R-Square	0.99999	
	Value	Standard Error
a0*	-2180.38381	6.22303
a1*	-102.07462	3.08907
a3*	0.8143	0.00906
b0*	10.55305	0.02167
b1*	0.35042	0.0106
T	273	0
	298	0

(c)

Equation	y=ln(x)+1/K*(a0+a1*x+a2*x^2+a3*x^3+a4*x^4+a5*x^5)+(b0+b1*x+b2*x^2)	
Adj.R-Square	0.99999	
	Value	Standard Error
a0*	-2349.36518	-2349.36518 6.58859
a1*	-150.64742	-150.64742 2.87796
a2*	10.15865	10.15865 1.15587
a3*	-1.25142	-1.25142 0.28905
a4*	0.21806	0.21806 0.02347
b0*	10.75786	10.75786 0.02293
b1*	0.42808	0.42808 0.00866
T	273	0
	298	0

Supplementary Figure 23 | Virial fitting of C_2H_2 (a), C_2H_4 (b) and C_2H_6 (c) adsorption data for **NPU-2** at 273 and 298 K.

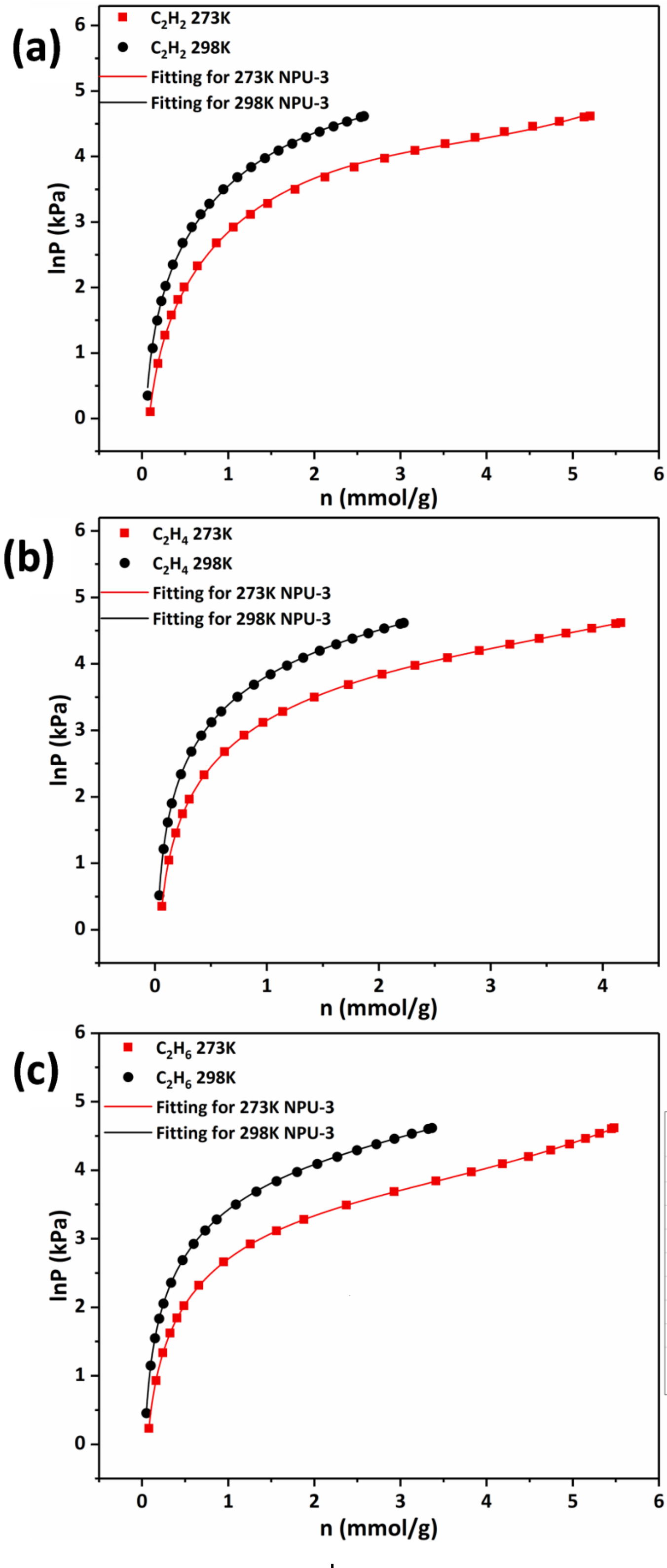

(a)

Equation	y=ln(x)+1/K*(a0+a1*x+a2*x^2+a3*x^3+a4*x^4+a5*x^5)+(b0+b1*x+b2*x^2)	
Adj.R-Square	0.99915	
	Value	Standard Error
a0*	-2407.19988	39.80758
a1*	166.01053	9.13213
a2*	-57.8786	4.59153
a3*	6.11205	0.6044
b0*	11.24422	0.13953
T	273	0
	298	0

(b)

Equation	y=ln(x)+1/K*(a0+a1*x+a2*x^2+a3*x^3+a4*x^4+a5*x^5)+(b0+b1*x+b2*x^2)	
Adj.R-Square	0.99998	
	Value	Standard Error
a0*	-2139.03642	10.34051
a1*	-16.65048	8.68031
a2*	-12.14026	0.17936
a3*	2.14026	0.17936
b0*	10.9537	0.0359
b1*	0.12773	0.02827
T	273	0
	298	0

(c)

Equation	y=ln(x)+1/K*(a0+a1*x+a2*x^2+a3*x^3+a4*x^4+a5*x^5)+(b0+b1*x+b2*x^2)	
Adj.R-Square	0.99997	
	Value	Standard Error
a0*	-2245.73791	10.68141
a1*	-88.11466	6.05942
a2*	-7.54286	0.66713
a3*	1.8929	0.08165
b0*	10.97857	0.03714
b1*	0.29495	0.01994
T	273	0
	298	0

Supplementary Figure 24 | Virial fitting of C_2H_2 (a), C_2H_4 (b) and C_2H_6 (c) adsorption data for **NPU-3** at 273 and 298 K.

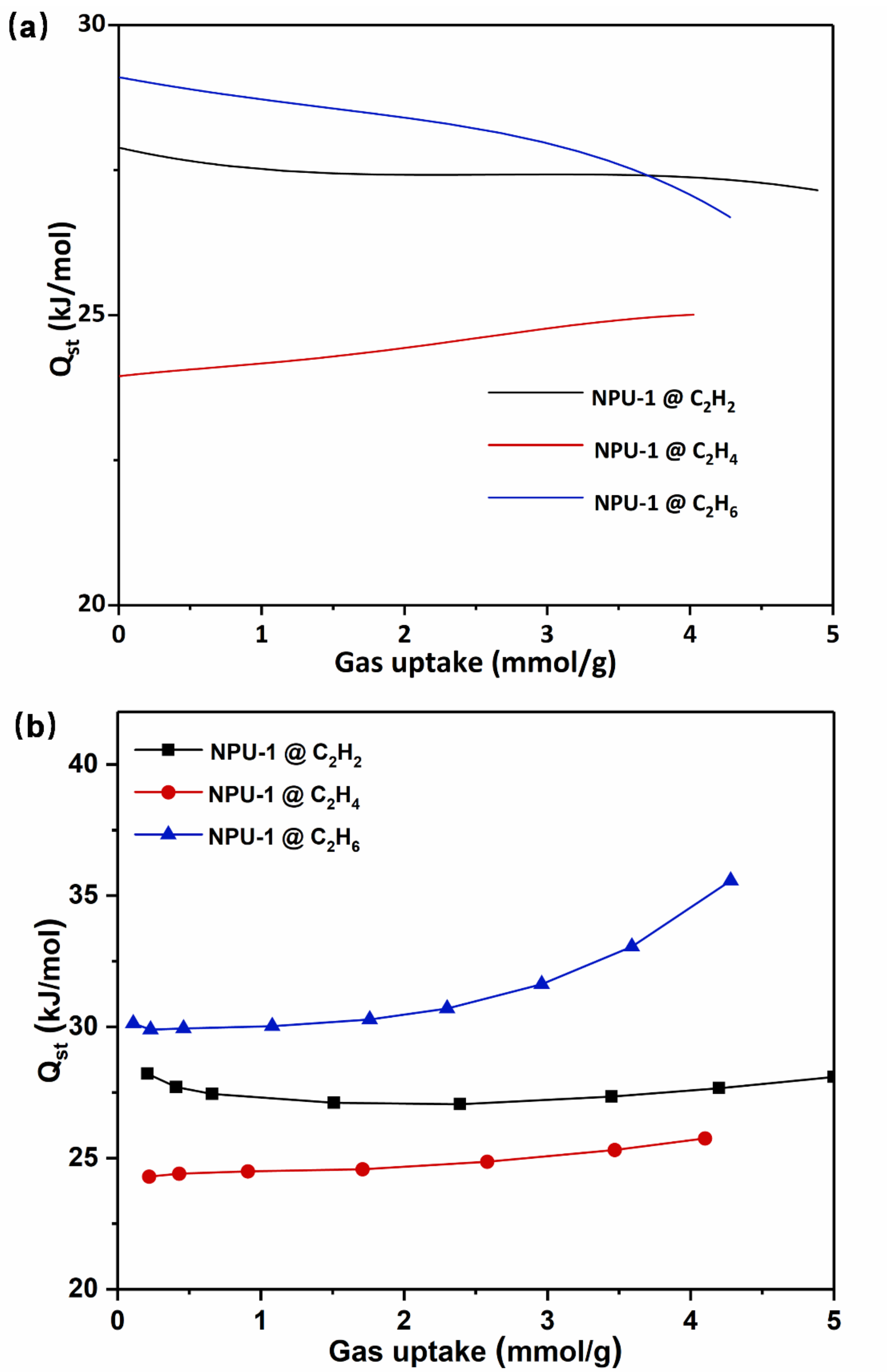

Supplementary Figure 25│ Gas adsorption enthalpy of activated **NPU-1** by virial equation fitting-based calculation (a) and direct calculation by Clausius-Clapeyron equation (b).

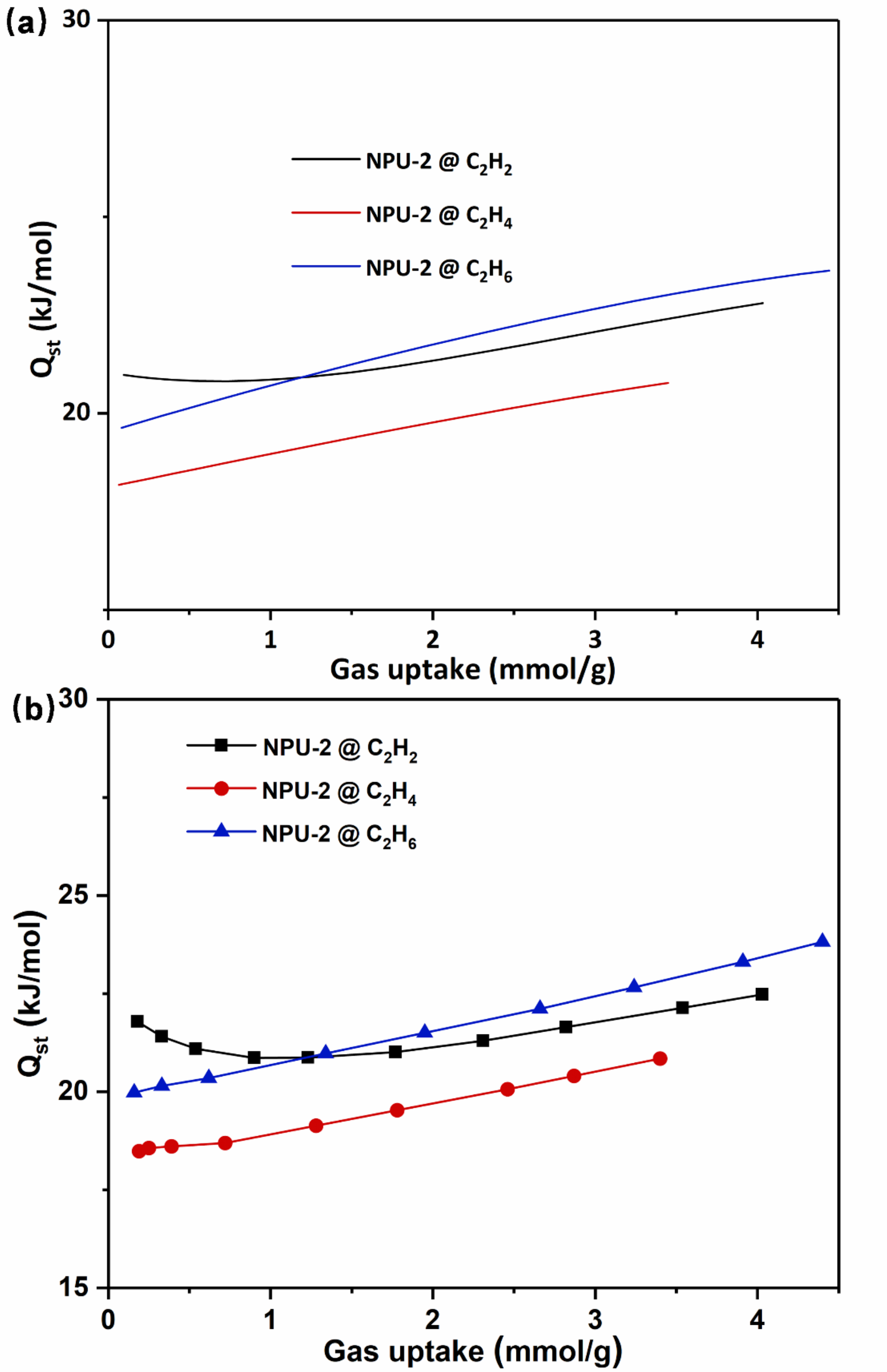

Supplementary Figure 26 | Gas adsorption enthalpy of activated **NPU-2** by virial equation fitting-based calculation (a) and direct calculation by Clausius-Clapeyron equation (b).

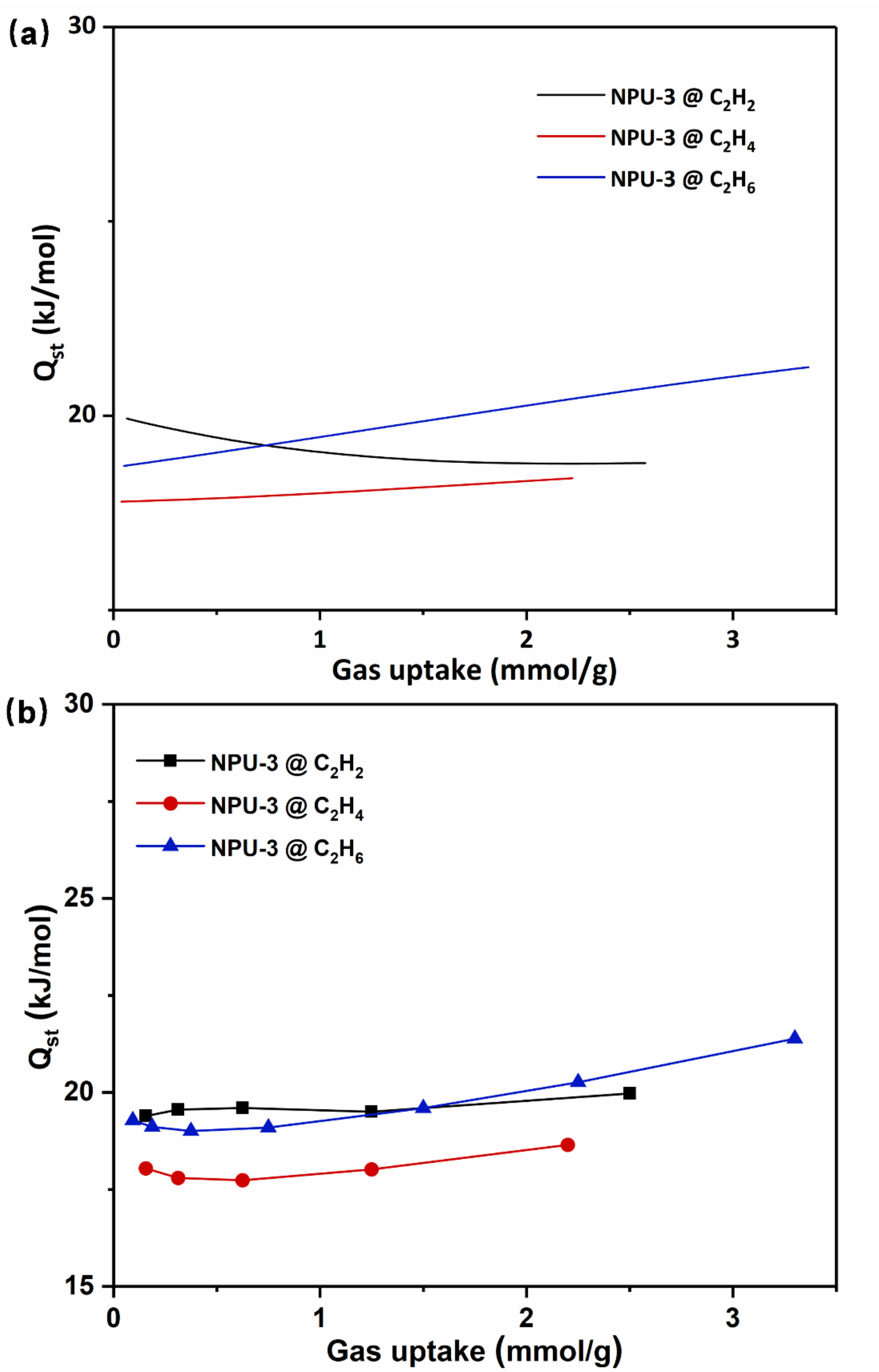

Supplementary Figure 27 | Gas adsorption enthalpy of activated **NPU-3** by virial equation fitting-based calculation (a) and direct calculation by Clausius-Clapeyron equation (b).

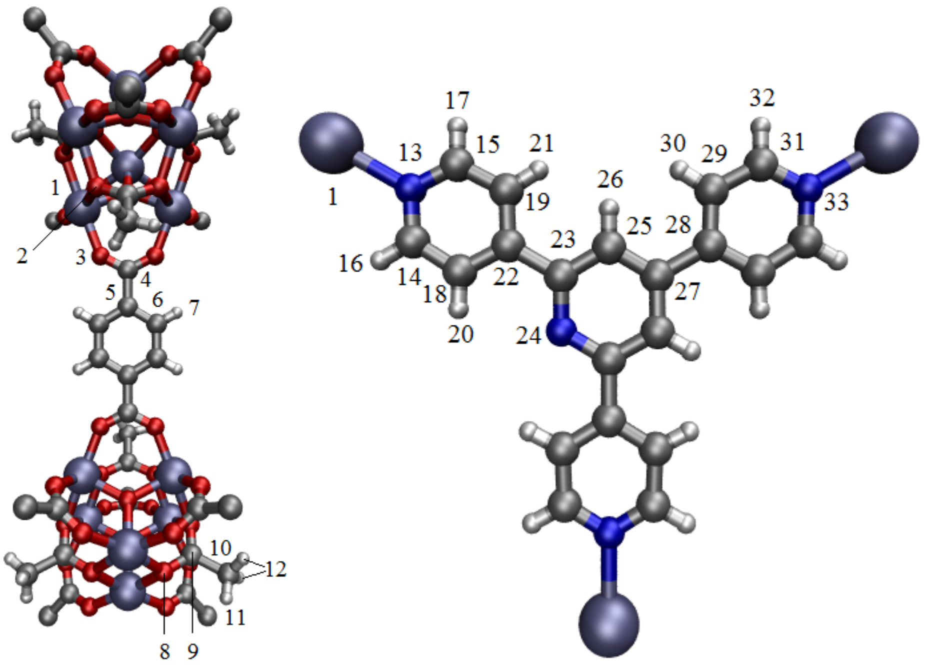

Supplementary Figure 28 | The chemically distinct atoms in **NPU-1** defining the numbering system corresponding to **Supplementary Table 2**. Atom colors: C = gray, H = white, N = blue, O = red, Mn = lavender.

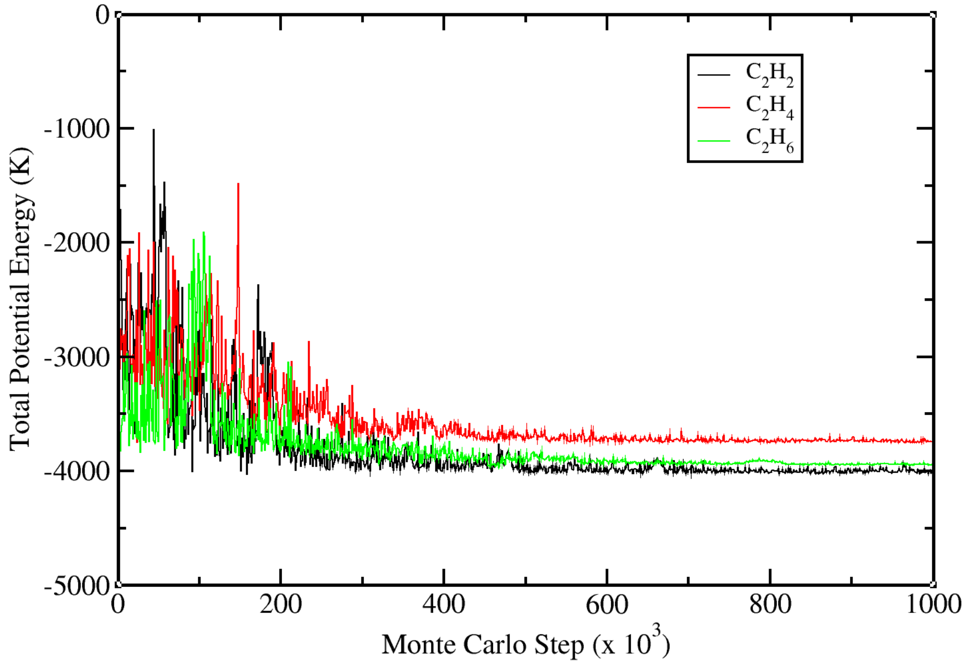

Supplementary Figure 29 | Calculated total potential energies (in K) as a function of MC steps from the SA calculations of a single molecule of C_2H_2 (black), C_2H_4 (red), and C_2H_6 (green) in **NPU-1**.

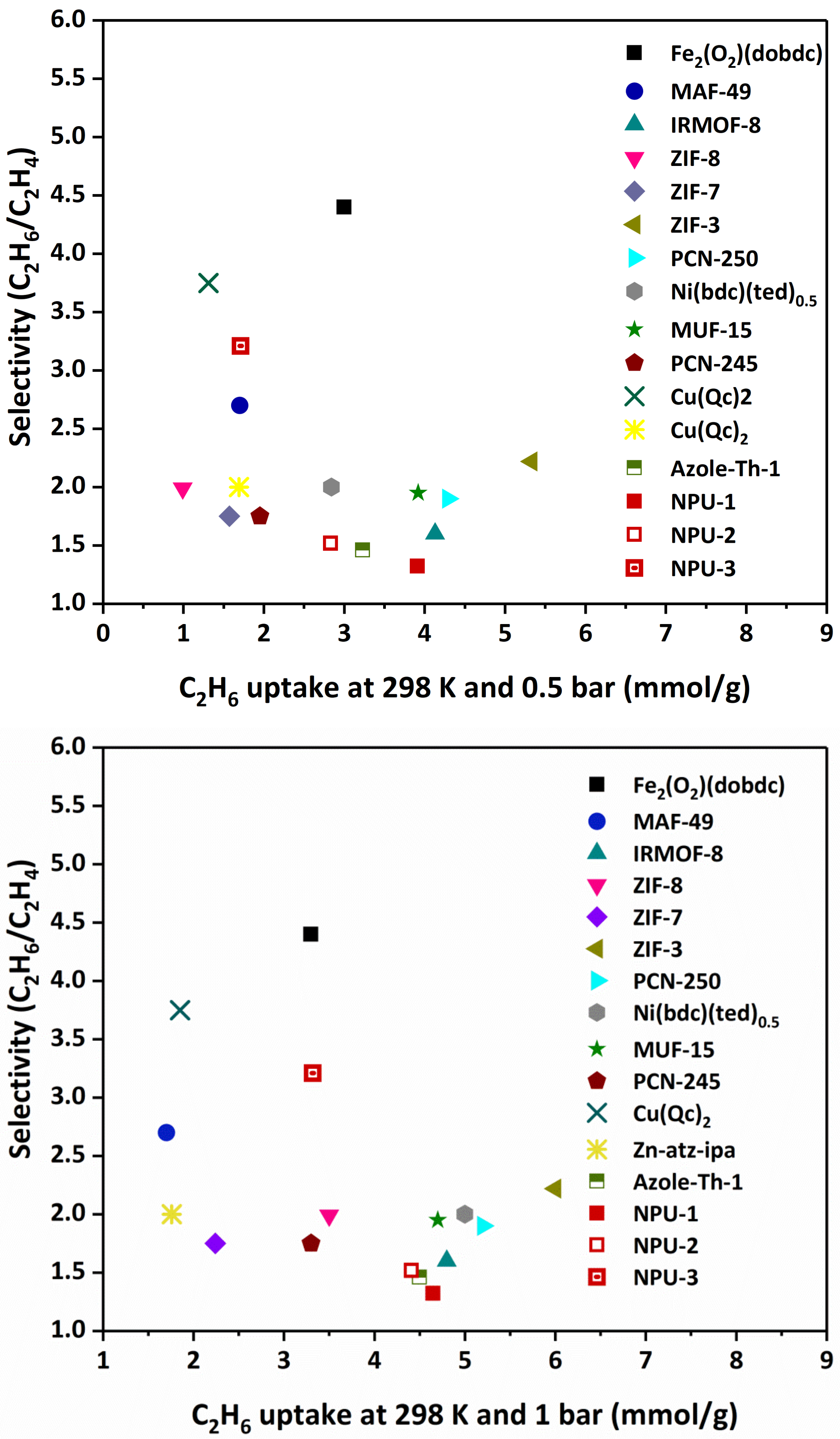

Supplementary Figure 30 | Comparison in C_2H_6 adsorption capacity and IAST selectivity of **NPU-1/2/3** with representative MOFs at 298K.[28-35]

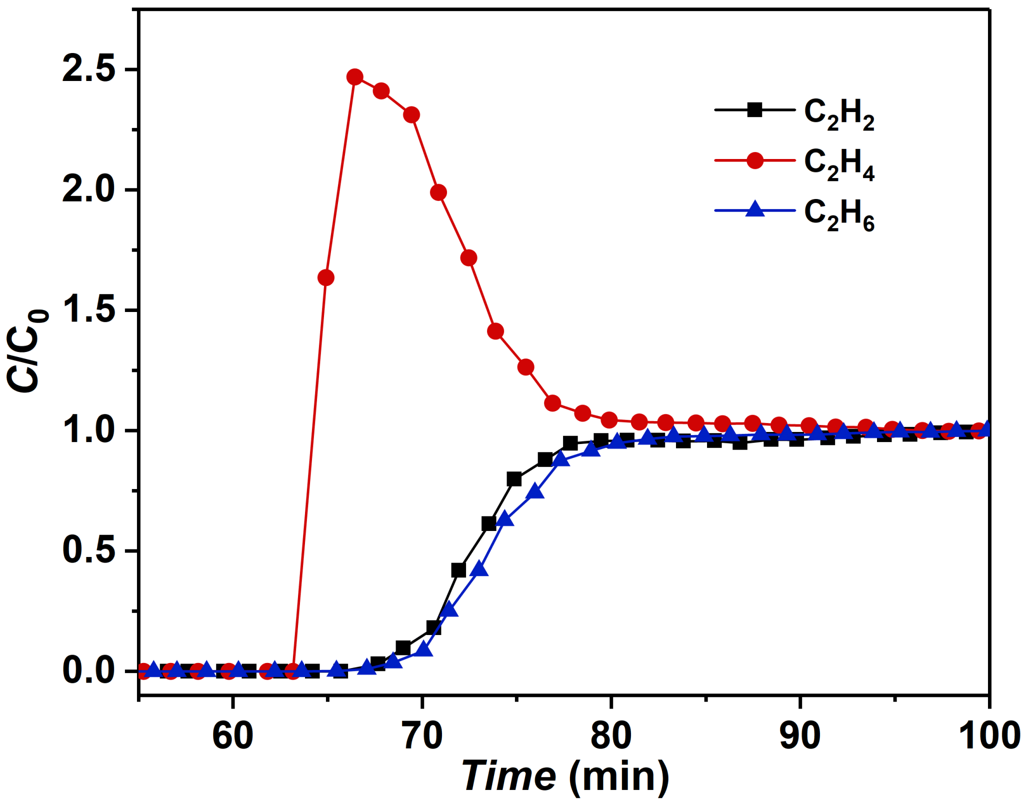

Supplementary Figure 31 | Experimental breakthrough curves at 298 K for $C_2H_2/C_2H_4/C_2H_6$ separation (1:1:1 mixture; total gas pressure of 100 kPa; total gas flow of 1.5 cm^3 min^{-1}) based on **NPU-1** column with 1.3 g sample.

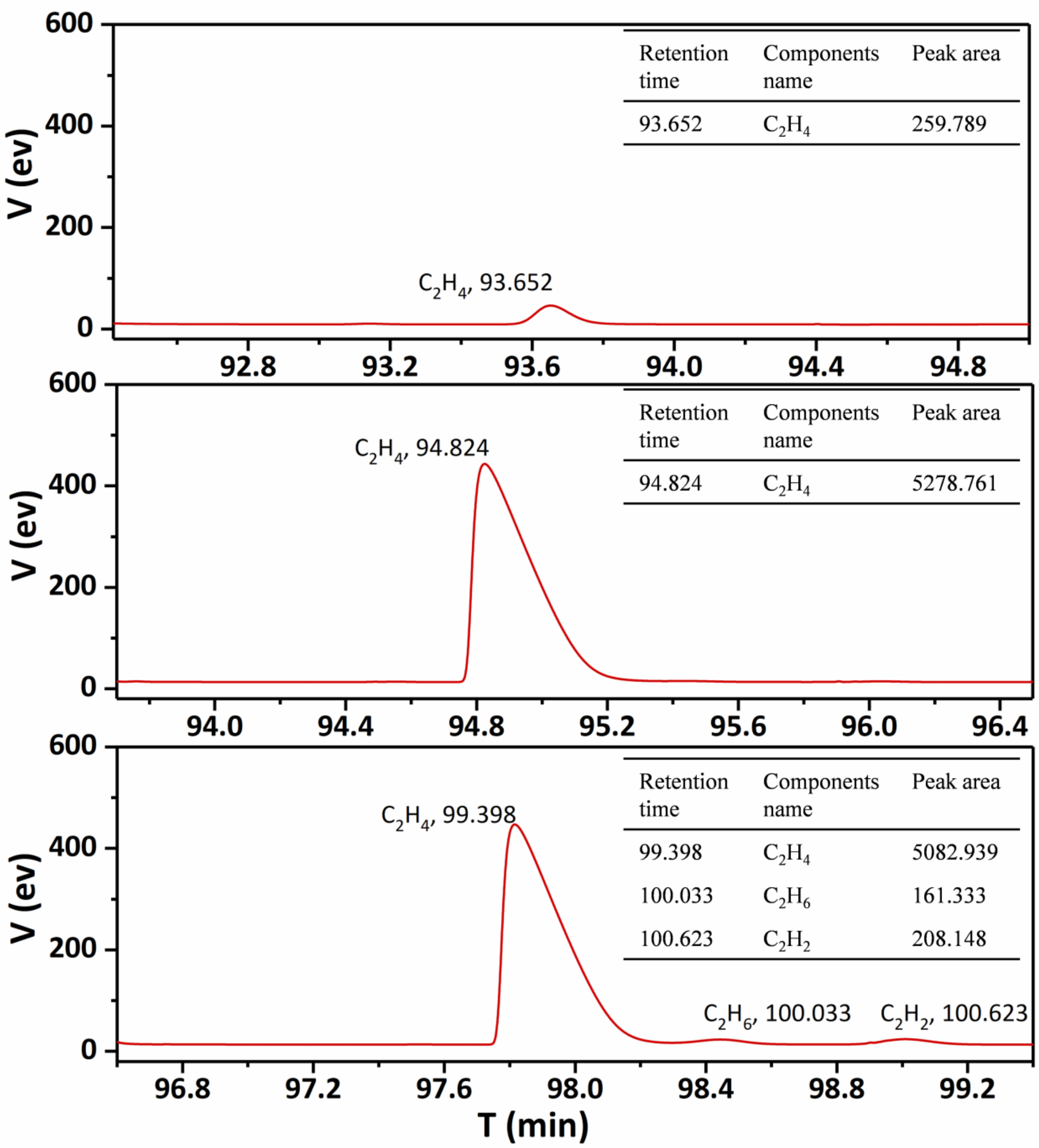

Supplementary Figure 32 | Time-dependent GC analysis of outlet gas concentration with **NPU-1** column (2.9 g) when passing $C_2H_2/C_2H_4/C_2H_6$ mixed gases (1:1:1 mixture; total gas pressure of 100 kPa; total gas flow of 2.1 cm^3 min^{-1}).

Supplementary Table 1 | Crystal data and structure refinements of **NPU-1**, **NPU-2** and **NPU-3**.

	**NPU-1**	**NPU-2**	**NPU-3**
Formula	$C_{70}H_{49}Mn_6N_8O_{20}$	$C_{84}H_{57}Mn_6N_6O_{20}$	$C_{88}H_{61}Mn_6N_8O_{20}$
Formula weight	1651.81	1799.99	1880.08
Temperature (K)	296(2)	296(2)	296(2)
Wavelength (Å)	0.71073	0.71073	0.71073
Crystal system	Hexagonal	Trigonal	Hexagonal
Space group	$P6_3/mmc$	$P\bar{3}1c$	$P6_3/mmc$
a (Å)	17.408(1)	17.369(5)	17.409 (1)
b (Å)	17.408(1)	17.369(5)	17.409(1)
c (Å)	21.848(2)	27.571(14)	32.506(1)
α (deg.)	90	90	90
β (deg.)	90	90	90
γ (deg.)	120	120	120
Volume (Å^3)	5733.5(7)	7204(5)	8531.8(10)
Z	2	2	2
F(000)	1670	1826	1910
Crystal size (mm)	0.36×0.35×0.18	0.33×0.27×0.26	0.35×0.28×0.27
Density (g cm^3)	0.957	0.830	0.732
R_{int}	0.1270	0.1521	0.0552
R_1 [$I > 2\sigma(I)$]	0.0573	0.1030	0.0446
wR_2 [$I > 2\sigma(I)$]	0.1533	0.2950	0.1321
R_1 (all data)	0.1042	0.1784	0.0607
wR_2 (all data)	0.1743	0.3385	0.1415
GOF	1.057	1.036	1.024

Supplementary Table 2 | Parameters for the chemically distinct atoms in NPU-1 that were used for the simulations in this work. Label of atoms correspond to Supplementary Figure 5.

Atom	Label	ε (K)	σ (Å)	q (e^-)	α° (Å^3)
Mn	1	6.54185	2.63795	1.3361	2.4875
O	2	30.19	3.118	–0.9249	0.8520
O	3	30.19	3.118	–0.8445	0.8520
C	4	52.84	3.75	1.0125	1.2886
C	5	35.25	3.55	–0.1087	1.2886
C	6	35.25	3.55	–0.1092	1.2886
H	7	15.11	2.42	0.1196	0.4138
O	8	30.19	3.118	–0.9631	0.8520
C	9	52.84	3.50	1.2812	1.2886
C	10	52.84	3.50	–0.4407	1.2886
H	11	15.11	2.50	0.0969	0.4138
H	12	15.11	2.50	0.1082	0.4138
N	13	34.72	3.261	–0.3427	0.97157
C	14	35.25	3.55	0.3021	1.2886
C	15	35.25	3.55	0.3160	1.2886
H	16	15.11	2.42	0.0402	0.4138
H	17	15.11	2.42	0.0391	0.4138
C	18	35.25	3.55	–0.4090	1.2886
C	19	35.25	3.55	–0.4670	1.2886
H	20	15.11	2.42	0.1471	0.4138
H	21	15.11	2.42	0.1645	0.4138
C	22	35.25	3.55	0.1857	1.2886
C	23	35.25	3.55	0.4109	1.2886
N	24	85.60	3.25	–0.5307	0.97157
C	25	35.25	3.55	–0.4589	1.2886
H	26	15.11	2.42	0.2498	0.4138
C	27	35.25	3.55	0.2007	1.2886
C	28	35.25	3.55	0.2366	1.2886
C	29	35.25	3.55	–0.4502	1.2886
H	30	15.11	2.42	0.1715	0.4138
C	31	35.25	3.55	0.3879	1.2886
H	32	15.11	2.42	0.0691	0.4138
N	33	34.72	3.261	–0.2504	0.97157

Supplementary Table 3 | Averaged classical potential energies (< *U* >) for C_2H_2, C_2H_4, and C_2H_6 localized at their global minimum in **NPU-1** according to SA calculations. The energies are shown in units of K and kJ mol^{-1}.

Adsorbate	< *U* > (K)	< *U* > (kJ mol^{-1})
C_2H_2	–3788.523	–31.50
C_2H_4	–3543.515	–29.46
C_2H_6	–3785.824	–31.48

Supplementary Table 4 | Summary of structural information and sorption data of **NPU-1/2/3** and representative MOFs.[36,37]

		NPU-1	NPU-2	NPU-3	TJT-100	Azole-Th-1
Pore size[a] (Å)		7.4	10.5	12.2	8.0	9.2
Porosity[b] (%)		50.8	56.6	61.9	47.5	50.1
N_2 uptake at 77 K and 1 bar (mmol/g)		13.74	19.01	23.84	11.16	11.80
BET surface (m^2g^{-1})		1396	1580	1834	890	982.97
Gas uptake at 298 K[c] (mmol/g)	C_2H_2	1.14 / 3.68 / 5.10	0.53 / 2.18 / 3.99	0.34 / 1.35 / 2.54	1.14 / 3.55 / 4.46	0.78 / 2.34 / 3.51
	C_2H_4	0.87 / 3.05 / 4.20	0.39 / 1.91 / 3.42	0.22 / 1.11 / 2.19	0.89 / 2.66 / 3.44	0.67 / 2.41 / 3.56
	C_2H_6	1.12 / 3.55 / 4.50	0.58 / 2.82 / 4.42	0.32 / 1.68 / 3.33	1.21 / 3.13 / 3.70	0.87 / 3.23 / 4.42
Gas uptake at 273K[d] (mmol/g)	C_2H_2	2.50 / 5.83 / 6.92	1.03 / 4.49 / 6.98	0.63 / 2.65 / 5.15	---	---
	C_2H_4	1.95 / 4.75 / 5.67	0.78 / 3.62 / 5.42	0.43 / 2.18 / 4.12	---	---
	C_2H_6	2.57 / 5.03 / 5.66	1.29 / 4.83 / 6.02	0.65 / 3.64 / 5.46	---	---
Q_{st}[d](kJ/mol)	C_2H_2	27.88	20.98	19.93	31.00	25.4
	C_2H_4	23.95	18.18	17.79	25.00	26.1
	C_2H_6	29.10	19.64	18.71	29.00	28.6
IAST selectivity[e]	C_2H_2 / C_2H_4	1.4*	1.25*	1.32*	1.8[×]	1.09*
	C_2H_6 / C_2H_4	1.32*	1.52*	3.21*	1.2[×]	1.46*

[a]: Calculated pore size based on the N_2 isotherm at 77 K according to the Horvath-Kawazoe model (pore geometry: cylinder); [b]: The accessible free volume estimated by Platon software without consideration of the solvent in the pore; [c]: Gas uptake at 298 K and 0.1/0.5/1.0 bar; [d]: Calculated gas adsorption enthalpy (Qst) at zero coverage. [e]: *C_2H_2 / C_2H_4 and C_2H_6 / C_2H_4 are 1/1 ratio. [×] C_2H_2 / C_2H_4 and C_2H_6 / C_2H_4 are 1/99 ratio.

References

1. Chen, K., Perry, J. J., Scott, H. S., Yang, Q. & Zaworotko, M. J., Double-walled pyr topology networks from a novel fluoride-bridged heptanuclear metal cluster. *Chem. Sci.* **6**, 4784 (2015).
2. Bruker, SMART and SAINT, Bruker AXS Inc., Madison, Wisconsin, USA (2012).
3. Bruker, SADABS, Bruker AXS Inc., Madison, Wisconsin, USA (2001).
4. Sheldrick, G. M., SHELXT-Integrated space-group and crystal-structure determination. *Acta Crystallogr. A.* **71**, 3 (2015).
5. Sheldrick, G. M., Crystal structure refinement with SHELXL. *Acta Crystallogr. C.* **71**, 3 (2015).
6. Farrugia, L. J., WinGX and ORTEP for Windows: an update. *J. Appl. Crystallogr.* **45**, 849 (2012).
7. Myers, A. L. & Prausnitz, J. M. Thermodynamics of mixed-gas adsorption. *AIChE. J.* **11**, 121 (1965).
8. Jorgensen, W. L., Maxwell, D. S. & Tirado-Rives, J. Development and testing of the OPLS all-atom force field on conformational energetics and properties of organic liquids. *J. Am. Chem. Soc*. **118**, 11225-11236 (1996).
9. Rappé, A. K., Casewit, C. J., Colwell, K. S., Goddard, W. A. & Skiff, W. M. U., A full periodic table force field for molecular mechanics and molecular dynamics simulations. *J. Am. Chem. Soc*. **114**, 10024-10035 (1992).
10. Valiev, M., Bylaska, E. J., Govind, N., Kowalski, K., Straatsma, T. P., Van Dam, H. J. J., Wang, D., Nieplocha, J., Apra, E., Windus, T. L., de Jong W. A., NWChem: A comprehensive and scalable open-source solution for large scale molecular simulations. *Comput. Phys. Commun*. **181**, 1477-1489 (2010).
11. Stevens, W. J., Basch, H. & Krauss, M. Compact effective potentials and efficient shared-exponent basis sets for the first- and second- row atoms. *J. Chem. Phys*. **81**, 6026-6033 (1984).
12. Hay, P. J & Wadt, W. R. Ab initio effective core potentials for molecular calculations. Potentials for the transition metal atoms Sc to Hg. *J. Chem. Phys*. **82**, 270-283 (1985).
13. LaJohn, L. A., Christiansen, P. A., Ross, R. B., Atashroo, T. & Ermler, W. C. Ab initio relativistic effective potentials with spin-orbit operators. III. Rb through Xe. *J. Chem. Phys*. **87**, 2812-2824 (1987).

14. Van Duijnen, P. T. & Swart, M. Molecular and Atomic Polarizabilities: Thole's model revisited. *J. Phys. Chem. A* **102**, 2399-2407 (1998).

15. Forrest, K. A., Franz, D. M., Pham, T. & Space, B. Investigating C_2H_2 sorption in α-[$M_3(O_2CH)_6$] (M = Mg, Mn) through theoretical studies. *Cryst. Growth Des.* **18**, 5342-5352 (2018).

16. Kirkpatrick, S., Gelatt, C. D. & Vecchi, M. P. Optimization by simulated annealing. *Science* **220**, 671-680 (1983).

17. Franz, D. M., Dyott, Z. E., Forrest, K. A., Hogan, A., Pham, T. & Space, B., Simulations of hydrogen, carbon dioxide, and small hydrocarbon sorption in a nitrogen-rich rht-metal-organic framework. *Phys. Chem. Chem. Phys.* **20**, 1761-1777 (2018).

18. Jones, J. E. On the determination of molecular fields. -II. From the equation of state of a gas. *Proc. R. Soc. Lond. Ser. A* **106**, 463-477 (1924).

19. Ewald, P. P. Die Berechnung optischer und elektrostantischer Gitterpotentiale. *Ann. Phys.*, **369**, 253-287 (1921).

20. Wells, B. A & Chaffee, A. L. Ewald summation for molecular simulations *J. Chem. Theory Comput.* **11**, 3684-3695 (2015).

21. Applequist, J., Carl, J. R. & Fung, K. Atom dipole interaction model for molecular polarizability. Application to polyatomic molecules and determination of atom polarizabilities. *J. Am. Chem. Soc.* **94**, 2952-2960 (1972).

22. Thole, B. Molecular polarizabilities calculated with a modified dipole interaction. *Chem. Phys.* **59**, 341-350 (1981).

23. Bode, K. A. & Applequist, J. A new optimization of atom polarizabilities in halomethanes, aldehydes, ketones, and amides by way of the atom dipole interaction model. *J. Phys. Chem.* **100**, 17820-17824 (1996).

24. McLaughlin, K., Cioce, C. R., Pham, T., Belof, J. L. & Space, B. Efficient calculation of many-body induced electrostatics in molecular systems. *J. Chem. Phys.* **139**, 184112 (2013).

25. Belof, J. L. & Space, B. *Massively Parallel Monte Carlo (MPMC)*. 2012, Available on GitHub. https://github.com/mpmccode/mpmc.

26. Franz, D. M., Belof, J. L., McLaughlin, K., Cioce, C. R., Tudor, B., Hogan, A., Laratelli, L., Mulcair, M., Mostrom, M., Navas, A., Stern, A. C., Forrest, K. A., Pham, T. & Space, B.,

MPMC and MCMD: Free high-performance simulation software for atomistic systems. *Adv. Theory Simul.* **2**, 1900113 (2019).

27. Zhai, Q., Bu, X., Mao, C., Zhao. X., Luke, D., Cheng, Y., Anibal J, R., Feng, P., An ultra-tunable platform for molecular engineering of high-performance crystalline porous materials. *Nat. Commun.* **7**, 13645 (2016).
28. Li, L.-B., Lin, R.-B., Krishna, R., Li, H., Xiang, S.-C., Wu, H., Li, J.-P., Zhou, W., Chen, B.-L., Ethane/ethylene separation in a metal-organic framework with iron-peroxo sites. *Science* **362**, 443 (2018).
29. Wu, Y., Chen, H., Liu, D., Qian, Y. & Xi, H., Adsorption and separation of ethane/ethylene on ZIFs with various topologies: Combining GCMC simulation with the ideal adsorbed solution theory (IAST). *Chem. Eng. Sci.* **124**, 144 (2015).
30. Liao, P., Zhang, W., Zhang, J. & Chen, X. Efficient purification of ethene by an ethane-trapping metal-organic framework. *Nat. Commun.* **6**, (2015).
31. Chen, D., Wang, N., Xu, C., Tu, G., Zhu, W., Krishna, R., A combined theoretical and experimental analysis on transient breakthroughs of C_2H_6/C_2H_4 in fixed beds packed with ZIF-7. *Microporous Mesoporous Mater.* **208**, 55 (2015).
32. Lv, D., Shi, R., Chen, R., Wu, Y., Wu, H., Xi, H., Xia, Q., Li, Z., Selective adsorption of ethane over ethylene in PCN-245: Impacts of interpenetrated adsorbent. *ACS Appl. Mater. Int.* **10**, 8366 (2018).
33. Chen, Y., Wu, H., Lv, D., Shi, R., Chen, Y., Xia, Q., Li, Z., Highly adsorptive separation of ethane/ethylene by an ethane-selective MOF MIL-142A. *Ind. Eng. Chem. Res.* **57**, 4063 (2018).
34. Lin, R., Wu, H., Li, L., Tang, X., Li, Z., Gao, J., Cui, H., Zhou, W., Chen, B., Boosting ethane/ethylene separation within isoreticular ultramicroporous metal-organic frameworks. *J. Am. Chem. Soc.* **140**, 12940 (2018).
35. Chen, K.-J., Madden, D.-G., Mukherjee, S., Pham, T., Forrest, K.-A., Kumar, A., Space, B., Kong, J., Zhang, Q.-Y., Zaworotko, M.-J., Synergistic sorbent separation for one-step ethylene purification from a four-component mixture. *Science* **366**, 241 (2019).
36. Hao, H.-G., Zhao, Y.-F., Chen, D.-M., Yu, J.-M., Tan, K., Ma, S.-Q., Chabal, Y., Zhang, Z.-M., Dou, J.-M., Xiao, Z.-H., Day, G., Zhou, H.-C., Lu, T.-B., Simultaneous trapping of C_2H_2 and C_2H_6 from a ternary mixture of $C_2H_2/C_2H_4/C_2H_6$ in a robust metal-organic framework for the purification of C_2H_4. *Angew. Chem. Int. Ed.* **57**, 16067 (2018).

37. Xu, Z.-Z., Xiong, X.-H., Xiong, J.-B., Krishna, R., Li, L.-B., Fan, Y.-L., Luo, F., Chen, B.-L., A robust Th-azole framework for highly efficient purification of C_2H_4 from a C_2H_4/C_2H_2/C_2H_6 mixture. *Nat. Commun.* **11**, (2020).
